# Pentadecapeptide BPC 157 as Therapy for Inferior Caval Vein Embolization: Recovery of Sodium Laurate-Post-Embolization Syndrome in Rats

**DOI:** 10.3390/ph16101507

**Published:** 2023-10-23

**Authors:** Ivan Maria Smoday, Ivan Krezic, Luka Kalogjera, Vlasta Vukovic, Helena Zizek, Marija Skoro, Katarina Kasnik Kovac, Hrvoje Vranes, Ivan Barisic, Suncana Sikiric, Sanja Strbe, Marijan Tepes, Katarina Oroz, Slavica Zubcic, Mirjana Stupnisek, Lidija Beketic Oreskovic, Ivana Kavelj, Luka Novosel, Matea Prenc, Sanja Barsic Ostojic, Ivan Dobric, Marko Sever, Alenka Boban Blagaic, Anita Skrtic, Mario Staresinic, Ivica Sjekavica, Sven Seiwerth, Predrag Sikiric

**Affiliations:** 1 Department of Pharmacology, School of Medicine, University of Zagreb, 10000 Zagreb, Croatia; ivansmoday1@gmail.com (I.M.S.); ivankrezic94@gmail.com (I.K.); lkalogjera9@gmail.com (L.K.); vukovic.vlasta1@gmail.com (V.V.); zizekhelena@gmail.com (H.Z.); kkasnik@gmail.com (K.K.K.); hrvoje.vranes@gmail.com (H.V.); inbarisic@gmail.com (I.B.); strbes@gmail.com (S.S.); mtepes@gmail.com (M.T.); oroz.kat@hotmail.com (K.O.); zubcic.slavica@gmail.com (S.Z.); stupnisek@gmail.com (M.S.); lidijabeketicoreskovic@gmail.com (L.B.O.); abblagaic@mef.hr (A.B.B.); 2 Department of Diagnostic and Interventional Radiology, University Hospital Centre, 10000 Zagreb, Croatia; skoro.marija13@gmail.com (M.S.); ivana.kavelj@gmail.com (I.K.); novosel0701@gmail.com (L.N.); prenc.matea2@gmail.com (M.P.); sanja.barsic@gmail.com (S.B.O.); ivica.sjekavica@zg.t-com.hr (I.S.); 3 Department of Pathology, School of Medicine, University of Zagreb, 10000 Zagreb, Croatia; suncanasikiric@gmail.com (S.S.); sven.seiwerth@mef.hr (S.S.); 4 Department of Surgery, School of Medicine, University of Zagreb,10000 Zagreb, Croatia; ivandobricmd@gmail.com (I.D.); dr.sever.marko@gmail.com (M.S.)

**Keywords:** post-embolization syndrome, general occlusion/occlusion-like syndrome, stable gastric pentadecapeptide BPC 157, therapy, rats

## Abstract

After inferior caval vein embolization therapy, post-embolization syndrome (sodium laurate 10 mg/kg, 0.1 mL into rat inferior caval vein, assessment at 15, 30, 60 min, prime lung lesions, thromboemboli occluding lung vessels), as a severe occlusion/occlusion-like syndrome, might be resolved as a whole by stable gastric pentadecapeptide BPC 157 therapy. At 5 min after laurate injection, stable gastric pentadecapeptide BPC 157 was implemented as therapy (10 µg/kg, 10 ng/kg intraperitoneally or intragastrically). As before, confronted with the occlusion of major vessel(s) or similar noxious procedures, such as rapidly acting Virchow triad circumstances, the particular effect of the therapy (i.e., collateral pathways activation, “bypassing vascular key”, i.e., direct blood flow delivery via activation of azygos vein) assisted in the recovery of the vessel/s and counteracted multiorgan failure due to occlusion/occlusion-like syndrome as a whole in the laurate-injected rats. Along with prime lung lesions and thromboemboli occluding lung vessels, post-embolization syndrome rapidly occurred peripherally and centrally as a shared multiorgan and vessel failure, brain, heart, lung, liver, kidney, and gastrointestinal tract lesions, venous hypertension (intracranial (superior sagittal sinus), portal, and caval), aortal hypotension, progressing thrombosis in veins and arteries and stasis, congested and/or failed major veins, and severe ECG disturbances. Whatever the cause, these were all counteracted, eliminated, or attenuated by the application of BPC 157 therapy. As recovery with BPC 157 therapy commonly and rapidly occurred, reversing the collapsed azygos vein to the rescuing collateral pathway might initiate rapid direct blood delivery and start blood flow reorganization. In conclusion, we suggest BPC 157 therapy to resolve further vascular and embolization injuries.

## 1. Introduction

This study attempts to resolve the consequences of inferior caval vein embolization in rats. In the studies of occlusion/occlusion-like syndromes, peripherally and centrally, that were counteracted by the stable gastric pentadecapeptide BPC 157 [1,2,3,4,5,6,7] therapy (i.e., rescuing the collateral pathway via direct blood flow delivery by activation of the azygos vein) [8,9,10,11,12,13,14,15,16,17,18,19,20,21,22,23,24], inferior caval vein embolization by sodium laurate application in rats can cause particular life-threatening circumstances, including prime lung lesions. Rapid post-embolization syndrome (i.e., thromboemboli occluding lung vessels) may instantly progress peripherally and centrally, with vascular and multiorgan failure (i.e., brain, heart, lung, liver, kidney, and gastrointestinal lesions), widespread thrombosis in arteries and veins, venous hypertension (intra-cranial (superior sagittal sinus), portal, caval), and aortal hypotension, as an advanced occlusion/occlusion-like syndrome and Virchow triad circumstances. Given the presence of prime lung lesions, this may be worse than occlusion/occlusion-like syndromes induced by the occlusion of peripheral [8,9,10,11,12,13,14] and central [15,16] major vessels, and similar noxious procedures [17,18,19,20,21,22,23,24], which were resolved as a whole by BPC 157 therapy [8,9,10,11,12,13,14,15,16,17,18,19,20,21,22,23,24].

To compete with and counteract the already advanced post-embolization syndrome, BPC 157 therapy was given intraperitoneally or intragastrically at 5 min after laurate had been injected into the inferior caval vein. Along with its role in counteracting occlusion/occlusion-like syndromes, peripherally and centrally, the beneficial peripheral and central effects on internal organs and brain lesions implicated BPC 157 in the functioning of the brain-gut and gut–brain axis [25]. Likewise, it has a particular beneficial action on striated, smooth, and heart muscle, resulting in therapeutic effects on heart disturbances, myocardial infarction, heart failure, pulmonary hypertension, arrhythmias, and thrombosis counteraction [26,27]. Its therapeutic effects in eye pharmacology (i.e., intraocular pressure, glaucoma, retinal ischemia) were recently reviewed [28]. In general, its special cytoprotective background (and, thereby, its beneficial pleiotropic therapeutic effects) that may be further extended in practice (i.e., very safe, lethal dose (LD1) not achieved in toxicology studies, used in ulcerative colitis phase II) has also been reviewed [6,29]. Note that, acting as a cytoprotective mediator that is native and stable in human gastric juice (note: the cytoprotection concept originated in the stomach), BPC 157 may be easily applied, including the per-oral route [1,2,3,4,5,6,25,26,27,28,29]. It was suggested that BPC 157 as such a peptide [6,29] might actively upgrade Robert’s (epithelium protection) [30] and Szabo’s (endothelium protection) [31] stomach cytoprotection concept. Acting as a cytoprotective mediator, it can translate the original cytoprotective maintenance of gastrointestinal mucosal and endothelial integrity to other tissue therapies [6,29], as the cytoprotection concept posits for cytoprotective agents’ pleiotropic activity [30,31]. In such a way, upgrading endothelium function leads to upgraded minor vessels that may compensate for the function of failed major vessels, activating collateral pathways and re-establishing reorganized blood flow [6,29].

BPC 157’s activation of the rescuing collateral pathways is an additional highlight and advantage, both theoretically and practically, for particular antithrombotic treatment in rats with prime lung lesions. As BPC 157 does not affect coagulation pathways [32,33,34,35], its particular antithrombotic evidence is raised from the particular method of counteraction (activation of the collateral pathways) of occlusion/occlusion-like syndromes [8,9,10,11,12,13,14,15,16,17,18,19,20,21,22,23,24]. This occurred equally in rats with major vessel occlusion [8,9,10,11,12,13,14,15,16], both peripherally [8,9,10,11,12,13,14] and centrally [15,16], and who underwent similar procedures that all severely impaired endothelium function [17,18,19,20,21,22,23,24]. There, given the progressively obstructed vessels due to embolization, the activation of the collateral pathways by BPC 157 therapy has so far demonstrated, focusing on small vessels [8,9,10,11,12,13,14,15,16,17,18,19,20,21,22,23,24], that the activation of azygos vein direct blood flow delivery may be a particular rescuing pathway to counteract all noxious chains of events that otherwise irreparably occur after the embolization of the inferior caval vein. An illustrative upgrading of the vessels occurred in glaucomatous rats (cauterization of three of four episcleral veins to induce severe rat glaucoma) through one episcleral vein by BPC 157 therapy, and the course of glaucoma could be both prevented and reversed, and intraocular pressure normalized [14,28]. Likewise, the activated azygos vein and direct blood flow delivery, also known as the “bypassing key”, rapidly triggered by the stable gastric pentadecapeptide BPC 157’s strong therapeutic effect, may be responsible for the consistent counteraction of occlusion/occlusion-like syndromes [6,29]. There, in rats with prime lung lesions, this may be particularly important. Namely, besides severe lung lesions (hemorrhage), based on previous studies [8,9,10,11,12,13,14,15,16,17,18,19,20,21,22,23,24], worse occlusion/occlusion-like syndrome cause–consequence lesion progression could be observed in: the brain (intracerebral and intraventricular hemorrhage); heart (congestion, infarctions, severe arrhythmias); liver, kidney, and gastrointestinal tract congestion; venous hypertension (intracranial (superior sagittal sinus), portal and caval); and aortal hypotension. Widespread thrombosis in veins and arteries [8,9,10,11,12,13,14,15,16,17,18,19,20,21,22,23,24], stasis, and major vessels failure (congested inferior caval and superior mesenteric vein, collapsed azygos vein) [8,9,10,11,12,13,14,15,16,17,18,19,20,21,22,23,24], peripherally and centrally advanced Virchow triad circumstances. BPC 157 therapy attenuated/eliminated all of these disturbances, and in particular, Virchow triad circumstances were fully reversed given both thrombosis and hemorrhage largely abrogated. In addition, there is its special interaction with nitric oxide (NO)-systems [36,37,38,39] as a whole supporting this vascular recovery potential, through the “bypassing key” activation of the collaterals depending on the given injury [8,9,10,11,12,13,14,15,16,17,18,19,20,21,22,23,24]. In a series of very distinctive disturbances models, as recently reviewed, BPC 157 induced the release of the NO on its own [36,37,38,39], counteracted the adverse effect of NOS-blockade (i.e., hypertension) or NOS-over-stimulation (i.e., hypotension), maintained thrombocytes function [32,33,34,35,36,37,38] and many molecular pathways [40,41,42,43,44,45,46,47,48,49], and controlled vasomotor tone and the activation of the Src-Caveolin-1-eNOS pathway [40,41]. There, in the vessel wall, Fourier transform infrared spectroscopy showed that the BPC 157 therapy produced an instant and rapid change in the lipid contents and protein secondary structure conformation that may support vessel function in even the worst circumstances [50].

Therefore, after inferior caval vein embolization, rapid post-embolization syndrome such as an occlusion/occlusion-like syndrome with prime lung lesions would rapidly progress pleiotropically with a cause–consequence course [8,9,10,11,12,13,14,15,16,17,18,19,20,21,22,23,24], both peripherally and centrally, altogether leaving the body unable to re-establish blood flow. Progressing endothelium dysfunction means the growing incapability of minor vessels to adapt, unable to substitute the function of disabled major vessels, and the concurrent progress of arrhythmias [8,9,10,11,12,13,14,15,16,17,18,19,20,21,22,23,24]. Consequently, these would contribute to the particular downhill course of inferior caval vein embolization, rapid post-embolization syndrome, and prime lung lesion and thromboemboli occluding lung vessels in laurate-induced occlusion/occlusion-like syndrome. So far, in a quite complex procedure (i.e., femoral artery clamping), sodium laurate intra-arterial application is a known method for the thromboangiitis obliterans in rats, mimicking Buerger disease in humans [51,52]. Thereby, the sodium laurate intravenous administration is suitable as soap embolus to be applied in the infrarenal inferior caval vein, and thereby to induce the prime lung lesions and thromboemboli occluding lung vessels, particular post-embolization syndrome, peripherally and centrally as a particular vascular and multiorgan failure occlusion/occlusion-like syndrome that, as above described, is clearly responsive to the application of BPC 157 therapy [8,9,10,11,12,13,14,15,16,17,18,19,20,21,22,23,24]. Of note for the consideration of pulmonary embolisms [53], although it is associated with high mortality, so far, particular vascular and multiorgan failure occlusion/occlusion-like syndrome [8,9,10,11,12,13,14,15,16,17,18,19,20,21,22,23,24] has remained not included in the pulmonary embolism modeling most often used to study the anticoagulant [54,55,56] and antiplatelet [57,58,59] activity of pharmacological substances.

Thus, confronted with progressing embolization and severely impacted lungs, and occlusion/occlusion-like syndrome, this therapy application might be used as an essential rapid effect that works as a general defensive response. The final BPC 157 argument might be, as mentioned before, its easy application (being stable and native in human gastric juice for more than 24 h) and safety (i.e., in an ulcerative colitis trial, it was found to be safe without adverse effects, and a lethal dose (LD1) was not achieved in toxicology studies) (for review, see i.e., [1,2,3,4,5,6,25,26,27,28,29]). Consequently, we used both intraperitoneal and intragastric regimens. This advantage may indicate its particular therapeutic potential in post-embolization syndrome using both intragastric and intraperitoneal applications. In addition, µg- and ng-dose ranges, shown to be consistently effective in previous occlusion/occlusion-like syndromes [8,9,10,11,12,13,14,15,16,17,18,19,20,21,22,23,24] and are supposed to be effective in these aggravated conditions as well, were consistently used.

## 2. Results

Commonly, sodium laurate inferior caval vein embolization, unless BPC 157 therapy is given, means rapid post-embolization occlusion/occlusion-like syndrome, peripherally and centrally, with prime lung lesions and thromboemboli occluding lung vessels, and even overwhelming occlusion/occlusion-like syndromes induced by the major vessel occlusion or similar noxious procedures (e.g., endothelium damaging agent application, myocardial infarction, acute pancreatitis, intra-abdominal hypertension) [8,9,10,11,12,13,14,15,16,17,18,19,20,21,22,23,24].

As a highlight (i.e., counteraction of prime lung lesion and thromboemboli occluding lung vessels), all of the BPC 157-treated rats presented rapid and then sustained activation of the azygos vein, enabling direct blood delivery that might help to instantly break the injurious circle. As the common key finding, this is likely to be responsible for the prompt recovery effect. The confirmative proof of the concept appeared as its counteraction mechanism, with the adjacent adverse occlusion/occlusion-like syndrome as a whole being attenuated/eliminated. Although obtained in more demanding noxious conditions, the BPC 157 therapeutic effect was similar to the previous BPC 157 therapy of the mentioned occlusion/occlusion-like syndromes [8,9,10,11,12,13,14,15,16,17,18,19,20,21,22,23,24].

### 2.1. A Perilous Syndrome Occurred Peripherally and Centrally

#### 2.1.1. Blood Pressure Disturbances

With BPC 157 therapy, after laurate application, the prompt reduction of the blood pressure disturbances may show a cause–consequence relation, the beneficial effect going on peripherally (portal and caval hypertension and aortal hypotension were almost annihilated) as well as even more centrally (superior sagittal sinus hypertension was attenuated) (Table 1). Otherwise, if therapy was not given, the portal, caval, and even intracranial (superior sagittal sinus) hypertension, as well as the aortal hypotension, rapidly appeared and remained sustainably present until the end of the experiments.

#### 2.1.2. Thrombosis

**Figure 1:**
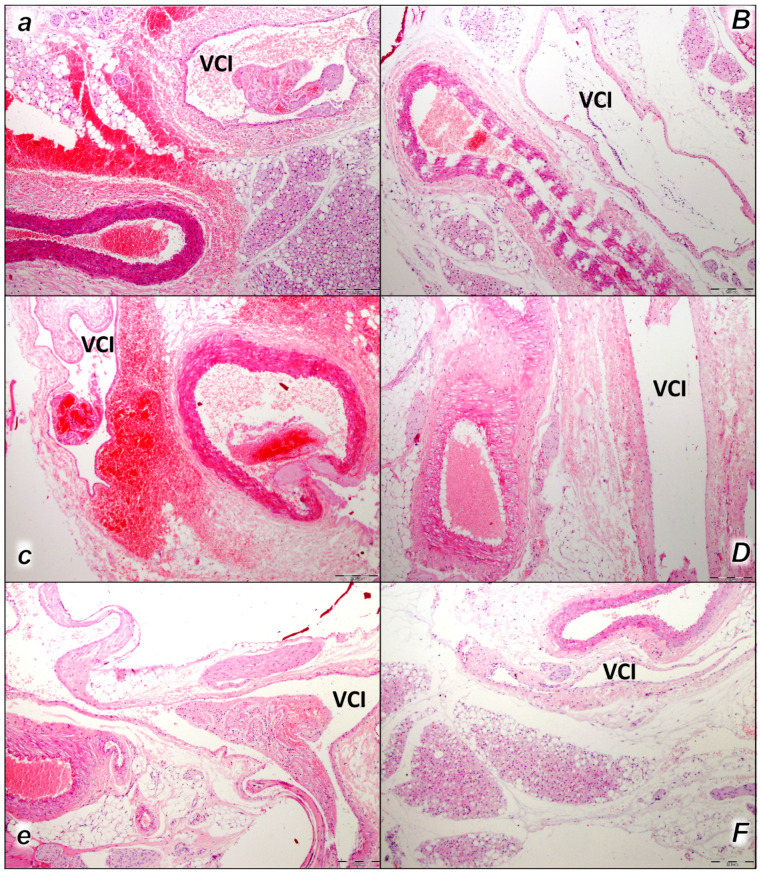
Thromboemboli presentation in vessels (***a****–**F***). In control rats, vascular thromboemboli presented within the lumen of large retroperitoneal vessels, abdominal aorta, and inferior vena cava (VCI) in all the time periods (15 min (***a***), 30 min (***c***), 60 min (***e***)) following laurate application into inferior caval vein. Thrombi consisted of fibrin and blood cells. In contrast, no thrombi were observed in treated animals. No thromboemboli were observed in BPC 157-treated group (15 min (***B***), 30 min (***D***), 60 min (***F***). (HE staining; magnification 100×; scale bar 200 μm).

**Figure 2:**
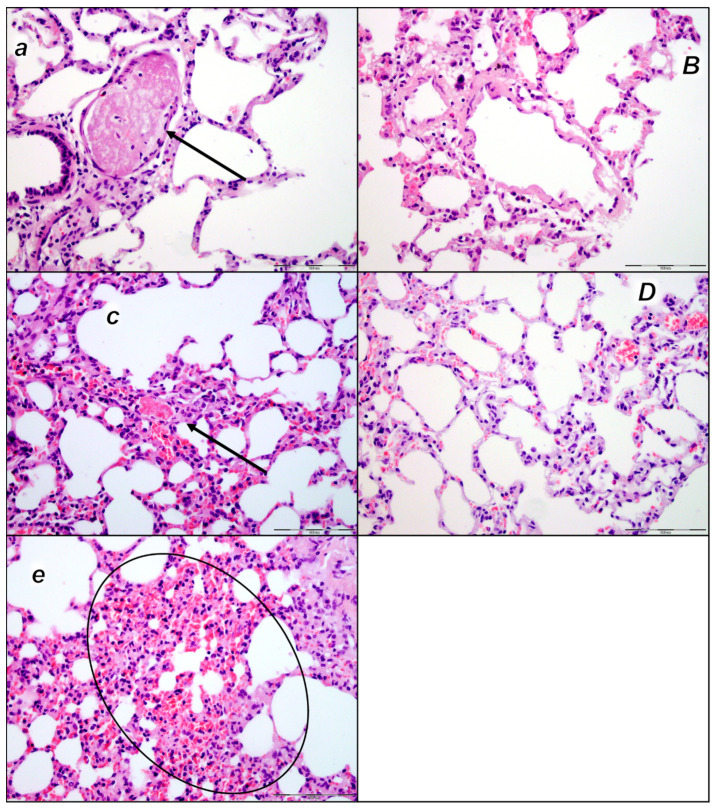
Thromboemboli presentation in lung (***a***–***e***). In control rats (*small italic letters*), thromboemboli (black arrow) were found within the lumen of medium and small lung and vascular vessels causing luminal occlusion, at two assessment time periods (30 min (***a***) and 60 min (***c***) following laurate application into inferior caval vein), with regional intraalveolar hemorrhagia (marked area) of lung parenchyma following thromboembolism after the time period of 60 min (***e***). Thromboemboli were absent in BPC 157-treated rats (*capital italic letters*) (30 min (***B***) and 60 min (***D***) following laurate application into the inferior caval vein. (HE staining; magnification 400×; scale bar 100 μm).

As the cause–consequence proof of the therapy’s effectiveness, thrombosis was promptly reduced by BPC 157 administration (gross assessment), peripherally and centrally (Table 1, Figure 1 and Figure 2). Otherwise, the noxious course was overwhelming, and widespread thrombosis progressed in veins and arteries (i.e., portal, caval vein, superior sagittal sinus, abdominal aorta).

This was along with microscopic thrombus assessment. Analyzing large retroperitoneal vessels, vascular thromboemboli were found in the control rats within the lumen of the abdominal aorta and inferior vena cava in all three assessment time periods (15 min, 30 min, and 60 min) (Figure 1). Thromboemboli consist of fibrin and blood cells. In lung tissue, control rats presented thromboemboli within the lumen of medium and small lung and vascular vessels causing luminal occlusion (Figure 2). They were found in two assessment time periods (30 min and 60 min following application), with regional intra-alveolar hemorrhagic features of lung parenchyma following thromboembolism after the time period of 60 min. Commonly, thromboemboli were absent in BPC 157-treated rats (Fisher exact probability test *p ˂ 0.05, at least* vs. *control*).

Only rarely were thromboemboli in the inferior vena cava found in BPC 157-treated rats, and most of these rats had no thromboemboli within the inferior vena cava at all (Fisher exact probability test * p ˂ 0.05, at least vs. *control*), while no thrombi were found in the abdominal aorta (Figure 1).

#### 2.1.3. Collateral Pathways, Blood Vessels, and Brain Gross Presentation

**Figure 3:**
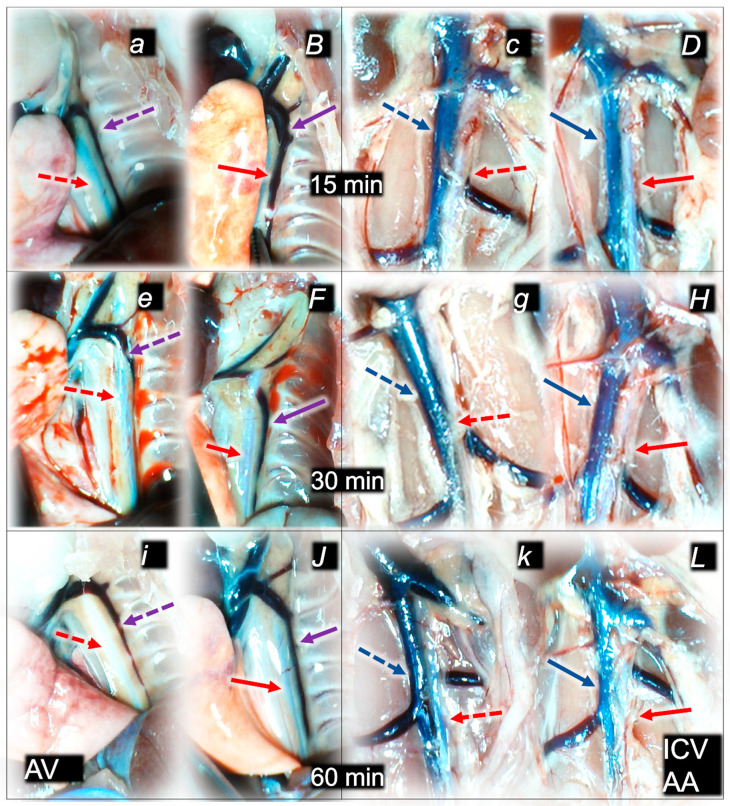
Illustrative presentation of azygos vein (AV) (violet arrows) and aorta (AA) in abdomen or thorax (red arrows) and inferior caval vein (blue arrows) (ICV) (***a***–***L***) in rats that received saline (control, small italic letters, dashed arrows) or BPC 157 therapy (capital italic letters, full arrows), immediately before sacrifice. Azygos vein and aorta at 15 min (***a***,***B***), 30 min (***c***,***D***), and 60 min *(**e***,***F***) following application of 0.1 mL of sodium laurate (10 mg/kg) into the inferior caval vein. Inferior caval vein and abdominal aorta at 15 min (***c***,***D***), 30 min (***g***,***H***), and 60 min (***k***,***H***) following application of 0.1 mL of sodium laurate (10 mg/kg) into the inferior caval vein.

**Figure 4:**
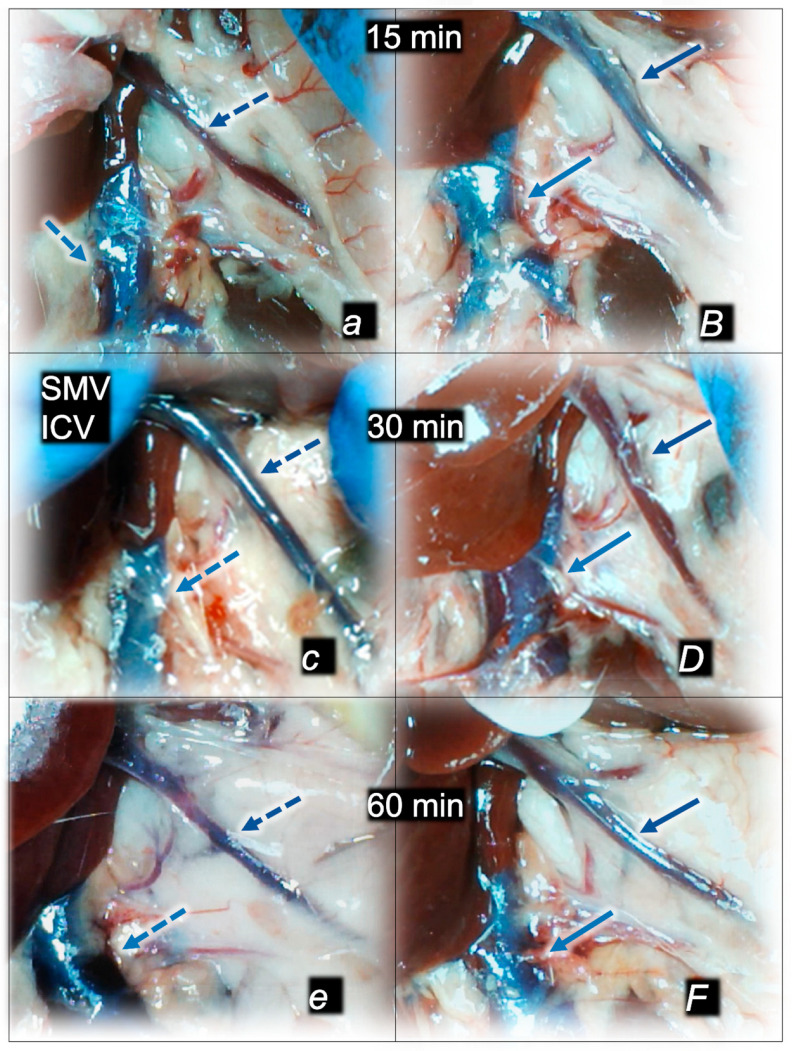
Illustrative presentation of superior mesenteric vein (SMV, dark blue arrows) and inferior caval vein (ICV, light blue arrows) (***a***,***B***,***c***,***D***,***e***,***F***) in rats that received 0.1 mL of sodium laurate (10 mg/kg) into the inferior caval vein, and then saline (control, small italic letters, dashed blue arrows) or BPC 157 therapy (capital italic letter, full blue arrows), immediately before sacrifice. Presentation at 15 min (***a***,***B***), 30 min (***c***,***D***), and 60 min (***e***,***F***) following application of 0.1 mL of sodium laurate (10 mg/kg) into the inferior caval vein.

**Figure 5:**
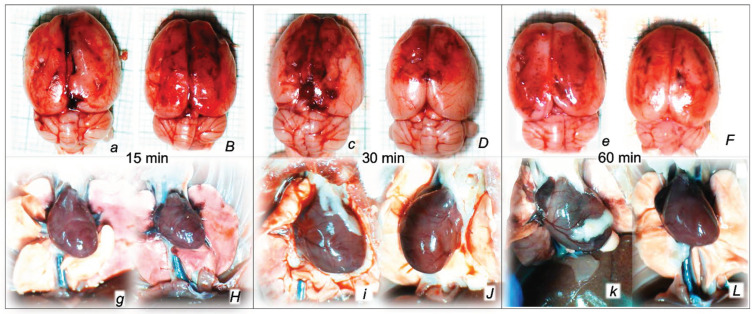
Illustrative presentation of brain (swelling) (***a***,***B***,***c***,***D***,***e***,***F***) and heart (dilatation) (***g***,***H***,***i***,***J***,***k***,***L***) in rats that in rats that received 0.1 mL of sodium laurate (10 mg/kg) into the inferior caval vein, and then saline (control, small italic letters) or BPC 157 therapy (capital italic letters). Presentation immediately after sacrifice (brain) or immediately before sacrifice (heart) at 15 min (***a***,***B***,***g***,***H***), 30 min (***c***,***D***,***i***,***J***) and 60 min (***e***,***F***,***k***,***L***) following application of 0.1 mL of sodium laurate (10 mg/kg) into the inferior caval vein.

Without therapy, post-embolization syndrome occurs with vessel congestion (superior mesenteric vein and inferior caval vein, due to the trapped volume, congested liver and lung), dilated heart, and collapsed vessels (not-functioning azygos vein) and swollen brain) (Figure 3, Figure 4 and Figure 5).

Without therapy, all of the laurate-injected rats converged to similar effects of continuous vascular failure, and recovery was not able to be spontaneously activated (Figure 3 and Figure 4). The failed collateral pathways presented in each of the given time points failed throughout the complete experimental period. Contrarily, advanced collateral pathways presentation consistently occurred with BPC 157 therapy (either intraperitoneal or intragastric) (Figure 3 and Figure 4). The particular vessel recruitment as a resolution for major vessel failure and stasis counteraction, peripherally and centrally, accords with blood pressure disturbances being attenuated/eliminated, and thrombosis being almost annihilated in veins and arteries, peripherally and centrally (Table 1). Thus, after laurate inferior caval vein embolization, BPC 157 therapy may fully reverse post-embolization syndrome.

Consequently, the particular effects of BPC 157 on the relative volume (Table 2) illustrate the activated defensive response as an immediate therapy effect. There was a reversal of the failed volume. Presentation of azygos vein collapse (as well as the abdominal aorta) was reversed and the azygos vein was reactivated (Figure 3). Likewise, there was a reversal of the increased relative volume (i.e., the superior mesenteric vein and inferior caval vein congestion, which BPC 157 might decrease) (Table 2, Figure 4).

As an immediate therapeutic effect, this could be illustrated by gross presentation in Figure 3, Figure 4 and Figure 5.

The essential point of the therapy was to reactivate the azygos vein, and, thereby, the collapsed volume of the azygos vein (as well as the abdominal aorta) was reversed (i.e., volume increased as the azygos vein was reactivated, thereby enabling direct blood delivery to occur) (Figure 3).

Likewise, we posit that this rescuing response results in the counteraction of the increased relative volume of the superior mesenteric vein and inferior caval vein (congestion), which were counteracted and reversed to normal vessel presentation (Figure 4).

The consistent outcome is that the presentation of these vessels (Figure 3 and Figure 4) and the heart (Figure 5) returns to close to normal vessel and heart presentation and close to normal functioning to re-establish blood flow (multiorgan lesions are largely attenuated) due to BPC 157 therapy. As further support, BPC 157 therapy induced a considerable change toward normal brain presentation and negative pressure values (Table 1 and Table 2) (i.e., brain swelling occurred with the increased intracranial (superior sagittal sinus) hypertension and increased volume (associated with considerable brain injuries) due to the laurate injection, which was reversed by BPC 157 (Figure 5).

#### 2.1.4. Heart and ECG Disturbances

Commonly, the laureate procedure implicates the prolongation of QTc intervals or PQ intervals, severe bradycardias, and therapeutic evidence in all BPC 157-treated rats (counteraction). The prolongation of QTc intervals or PQ intervals was regularly absent while bradycardia was attenuated. This occurred along with a counteraction of myocardial congestion (Table 3).

### 2.2. A Perilous Syndrome Occurred Peripherally

#### 2.2.1. Heart, Lung, Liver, Kidney, and Gastrointestinal Lesions

After regular laurate inferior caval vein embolization, similar considerable organ lesions indicate a failed common clue (i.e., venous hypertension (intracranial (superior sagittal sinus), portal, and caval) and aortal hypotension, progressed thrombosis, failed collateral recruitment, peripherally and centrally) and advanced post-embolization syndrome. Contrarily, it is likely that the reduced severity of lesions by BPC 157 therapy as an activated clue occurred as result of the immediate impact of the activated collateral pathway as part of the cause–consequence therapeutic course to counteract all of these disturbances.

#### 2.2.2. Heart

**Figure 6:**
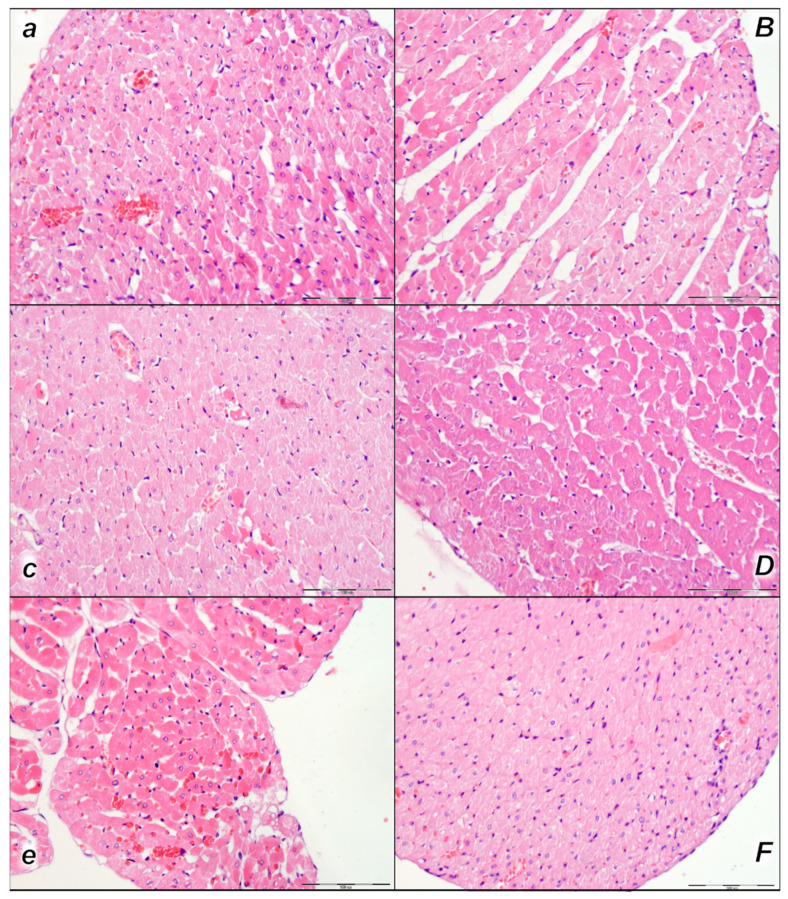
Heart, microscopy presentation (***a****–**F***). Pronounced congestion and dilatation of coronary arteries and their intramyocardial branches up to the subendocardial area in control rats (small italic letters) at 15 min (***a***), 30 min (***c***), and 60 min (***e***) after laurate application into inferior caval vein. No changes in BPC 157-treated rats (capital italic letters) were observed at 15 min (***B***), 30 min (***D***), and 60 min (***F***) after laurate application into the inferior caval vein. (HE staining; magnification 400×; scale bar 100 μm).

Histologic evaluation of representative myocardium tissue showed pronounced congestion and dilatation of coronary arteries and their intramyocardial branches up to the subendocardial area in control rats in all three assessment time periods (15 min, 30 min, and 60 min following laurate application). In BPC 157-treated rats, no or only mild congestion was observed (Table 4, Figure 6).

#### 2.2.3. Lung

**Figure 7:**
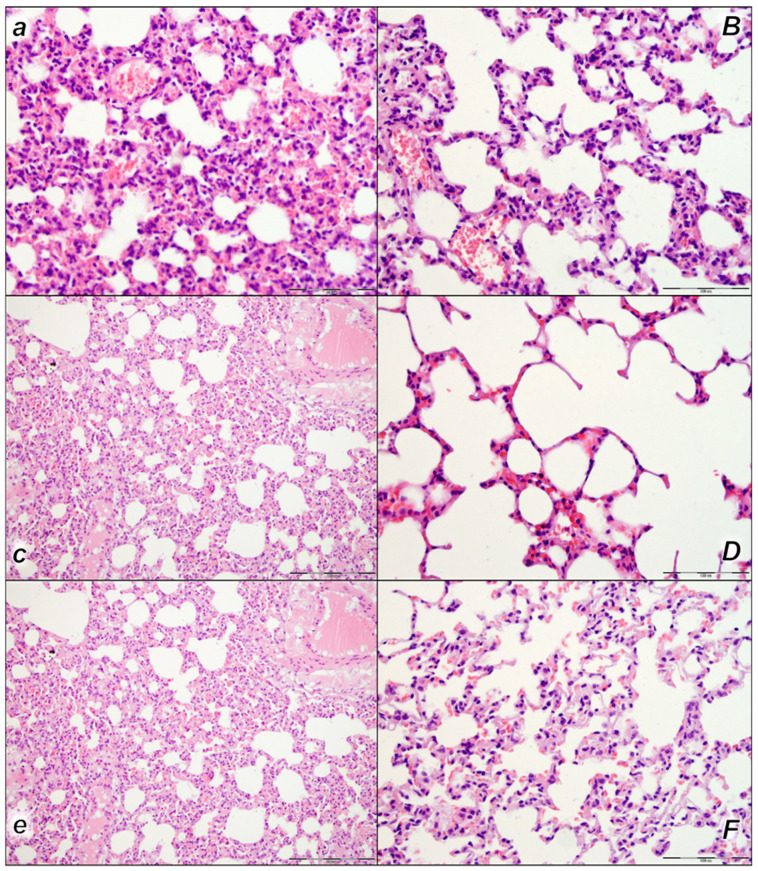
Lung, microscopy presentation (***a****–**F***). In the control rats, within-lung parenchyma thickening of the alveolar membranes was observed due to capillary congestion, pulmonary edema, and dilatation of larger blood vessels (small italic letters) at 15 min (***a***), 30 min (***c***), and 60 min (***e***) after laurate application into the inferior caval vein. No changes in BPC 157-treated rats were observed (capital italic letters) at 15 min (***B***), 30 min (***D***), and 60 min (***F***) after laurate application into the inferior caval vein. (HE staining; magnification 400×; scale bar 100 μm).

We noted, in the control rats, the thickening of the alveolar membranes due to capillary congestion, pulmonary edema, and dilatation of larger blood vessels in all three assessment time periods (15 min, 30 min, and 60 min following laurate application). In addition, focal intralveolar hemorrhage was found at 60 min following application. No changes were found in BPC 157-treated rats (Table 4, Figure 7).

#### 2.2.4. Liver

**Figure 8:**
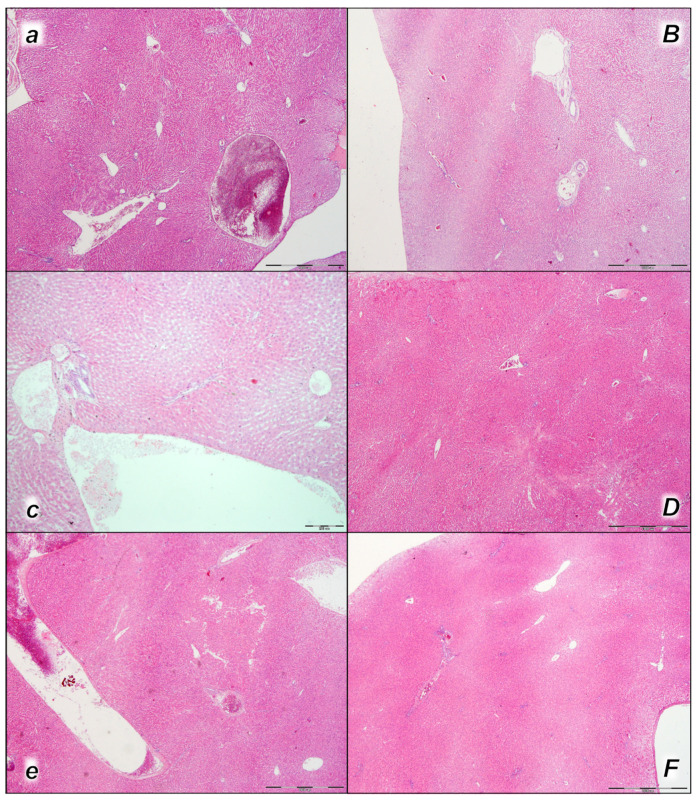
Liver, microscopy presentation (***a****–**F***). In liver parenchyma, pronounced dilatation of sinusoids and branches of the portal vein in portal tracts was found in liver tissue of control rats (small italic letters) at 15 min (***a***), 30 min (***c***), and 60 min (***e***) after laurate application into the inferior caval vein. No changes in BPC 157-treated rats (capital italic letters) were observed at 15 min (***B***), 30 min (***D***), and 60 min (***F***) after laurate application into the inferior caval vein. (HE staining; magnification 400×; scale bar 100 μm).

Pronounced dilatation of sinusoids and branches of the portal vein in portal tracts was found in the liver tissue of control rats in all three assessment time periods (15 min, 30 min, and 60 min following application). No changes were found in BPC 157-treated rats in the first two assessment time periods (15 min, and 30 min), and only mild congestion of liver parenchyma was found at 60 min following application (Table 4, Figure 8).

#### 2.2.5. Kidney

**Figure 9:**
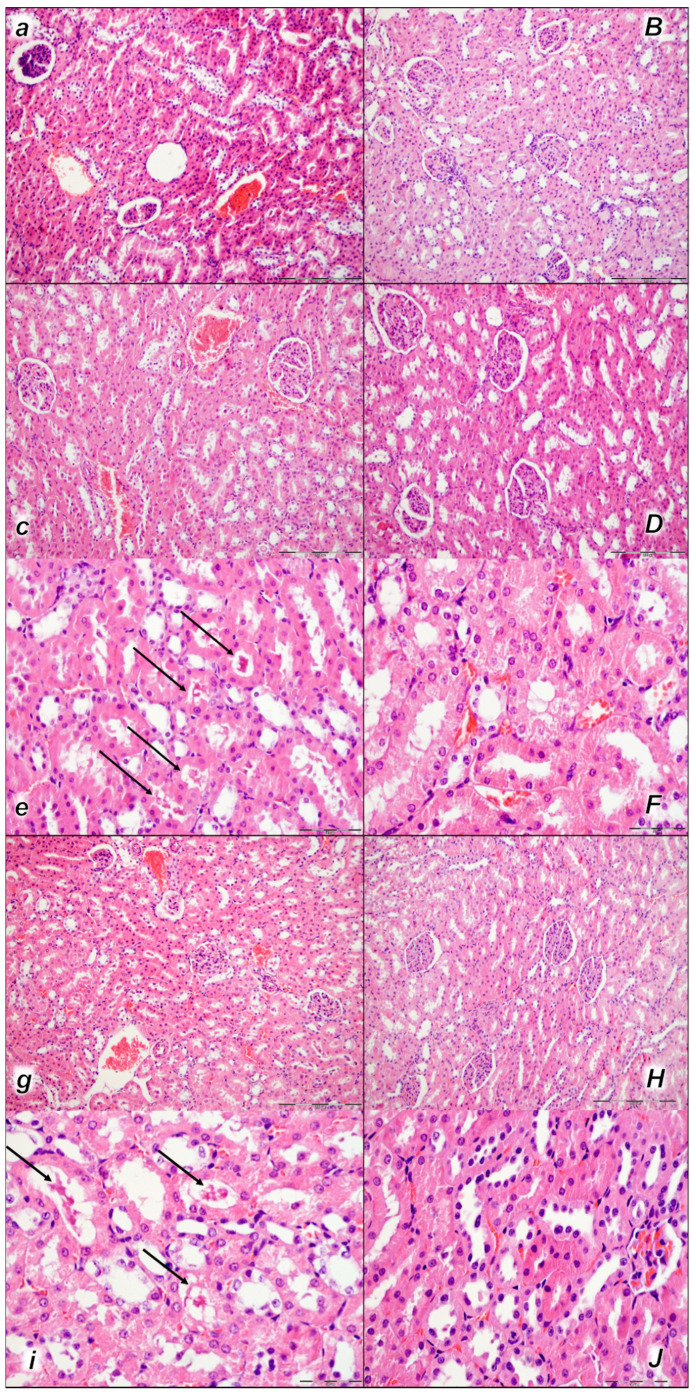
Kidney, microscopy presentation (***a***–***F***). After laurate application into the inferior caval vein control rats (small italic letters), moderate vascular congestion was shown in renal parenchyma, interstitial edema at 15 min (***a***), and moderate vascular congestion, and interstitial edema, and intratubular hyaline casts (black arrows) at 30 min (***c***,***e***) and at 60 min (***g***,***i***). No changes in BPC 157-treated rats (capital italic letters) were observed at 15 min (***B***), 30 min (***D***,***F***), and 60 min (***H***,***J***) after laurate application into the inferior caval vein. (HE staining; magnification 200×; scale bar 200 μm (***a***,***B***,***c***,***D***,***g***,***H***); magnification 600×; scale bar 50 μm (***e***,***F***,***i***,***J***)).

Moderate to marked vascular congestion and interstitial edema were found in control rats in all three assessment time periods (15 min, 30 min, and 60 min) following application. In addition, intratubular hyaline casts were found in control rats at 30 min and 60 min following application. No changes were found in BPC 157-treated rats (Table 4, Figure 9).

#### 2.2.6. Stomach, Small Intestine, and Colon Lesions

At 15 min, 30 min, and 60 min after laurate application into the inferior caval vein, the continuation of a noxious course without therapy is associated with hemorrhagic stomach lesions, marked congestion of submucosal blood vessels, and moderate dilatation of intramucosal blood vessels in the stomach and small intestinal and colonic wall. These were completely counteracted in BPC 157-treated rats (Table 4).

### 2.3. A Perilous Syndrome Occurred Centrally

#### 2.3.1. Brain Lesions, Cerebral and Cerebellar Cortex, Hypothalamus/Thalamus, and Hippocampus

**Figure 10:**
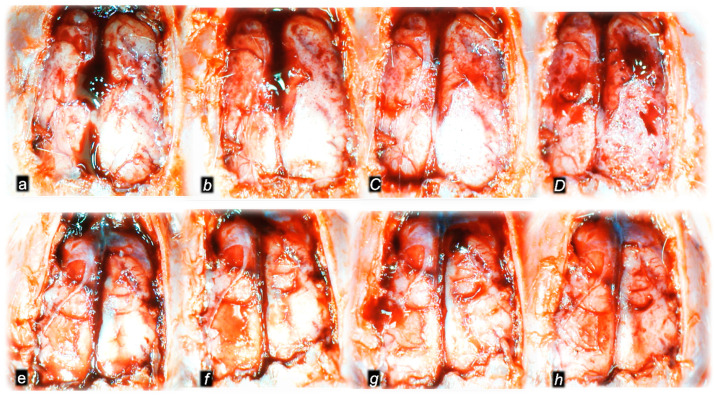
Gross brain presentation in healthy rats (normal small letters) and then after laureate application into inferior caval vein (italic letters), and subsequent application of the BPC 157 therapy (capital italic letters) or saline (small italic letters) (**a**–***h***). Brain presentation in normal healthy rats (**a**,**e**), brain swelling presentation immediately upon laurate application (***b***,***f***). Then, there was the opposite effect of therapy: decreased brain swelling immediately upon BPC 157 administration (***C***), decreased brain swelling in BPC 157-treated rats immediately before sacrifice (***D***), in contrast to further brain swelling immediately upon saline administration (***g***) and increased brain swelling in saline-treated rats immediately before sacrifice (***h***). A similar presentation was noted with both intragastric and intraperitoneal BPC 157 therapy.

**Figure 11:**
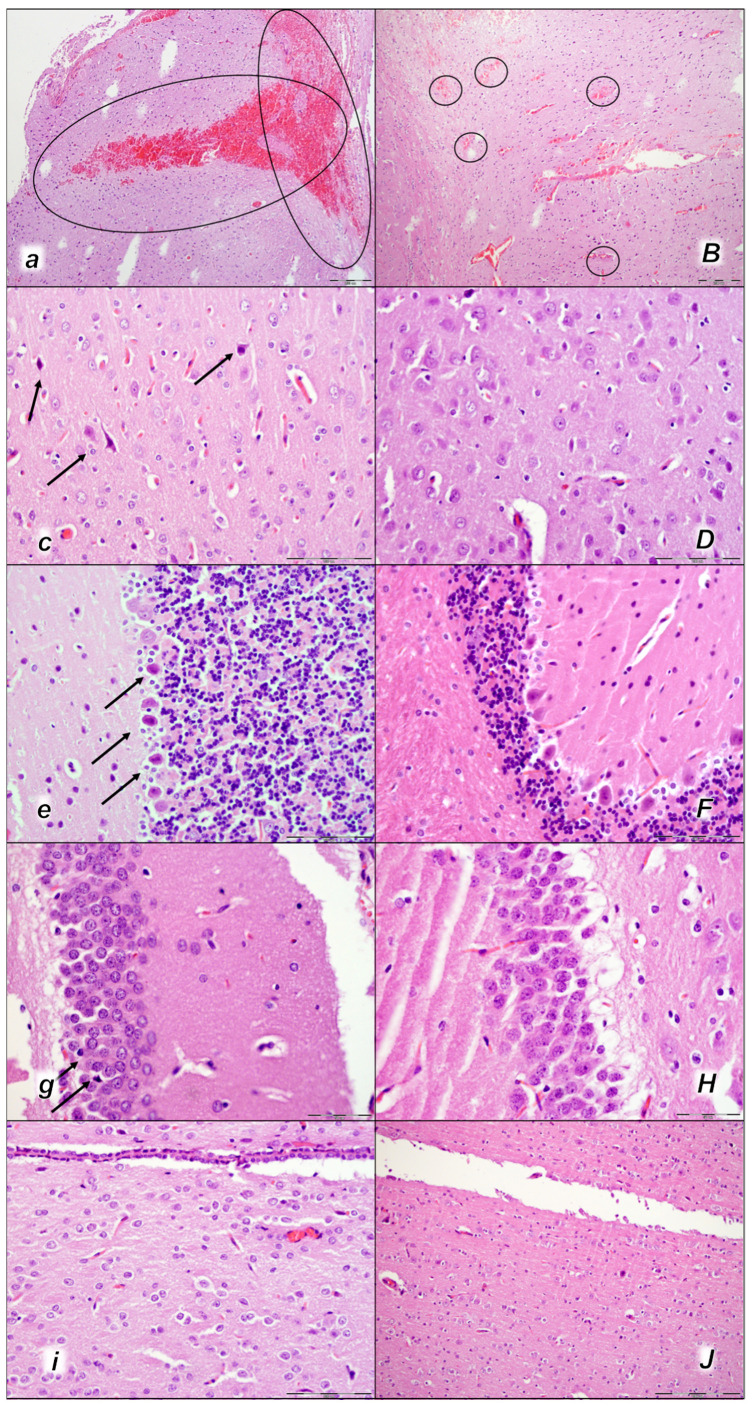
Brain neuropathological changes, 15 min following laurate application into the inferior caval vein, (***a****–**J***). In the control rats (small italic letters), a pronounced edema and congestion in the brain tissue were observed. Focal, pronounced, and deep intracerebral hemorrhage involving areas of brain tissue was observed, affecting areas of the neocortex, the corpus callosum, the amygdala, and the striatum in the brain tissue (***a***) (marked area). Mild neurodegenerative changes of the central nervous system, such as rare karyopyknotic cells affecting cerebral (***c***) and cerebellar (***e***) cortex, a karyopyknosis and degeneration of Purkinje cells of the cerebellar cortex, and karyopyknosis of cortical neurons, were observed (black arrows), including in the hypothalamus/thalamus (***g***) and hippocampus (***i***). In the BPC 157-treated rats (capital italic letters), mild edema and congestion in the brain tissue. Intracerebral hemorrhage was visible only within superficial layers of the neocortex (marked area) (***B***). No neurodegenerative changes of the central nervous system were observed in BPC 157 rats: in the cerebrum (***D***), cerebellum (***F***), hypothalamus/thalamus (***H***), and hippocampus (***J***). (HE staining; magnification 100×; scale bar 200 µm (***a***,***B***); magnification 400×; scale bar 100 μm (***c***–***J***)).

**Figure 12:**
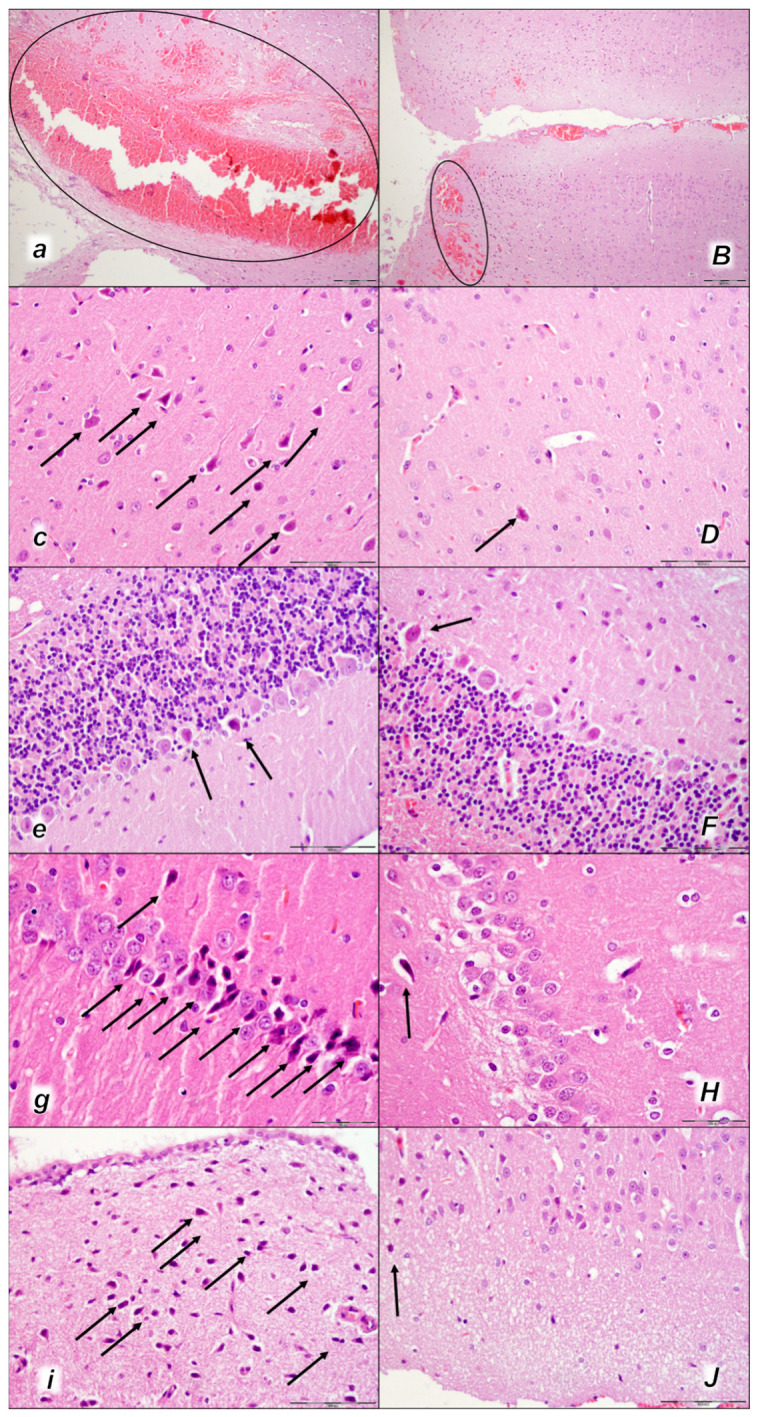
Brain neuropathological changes 30 min following laurate application into the inferior caval vein (***a****–**J***). In the control rats (italic small letters), a pronounced edema and congestion in the brain tissue were observed. Focal, pronounced, and deep intracerebral hemorrhage involving areas of brain tissue was observed, affecting areas of the neocortex, the corpus callosum, the amygdala, and the striatum in the brain tissue (***a***) (marked area). Moderate and severe neurodegenerative changes of the central nervous system, such as rare karyopyknotic cells affecting the cerebral (***c***) and cerebellar (***e***) cortex, a karyopyknosis and degeneration of the Purkinje cells of the cerebellar cortex, and karyopyknosis of cortical neurons, were observed (black arrows), as well as karyopyknosis of cortical neurons, hypothalamic neurons (***g***), and pyramidal cells of the hippocampus (***i***) (black arrows). In the BPC 157-treated rats (capital italic letters), only mild edema and congestion in the brain tissue were observed. Intracerebral hemorrhage was visible only within superficial layers of the neocortex (marked area) (***B***). BPC 157-treated rats presented no or only rare karyopyknotic cells in all four regions: the cerebrum (***D***), cerebellum (***F***), hypothalamus/thalamus (***H***), and hippocampus (***J***). (HE staining; magnification 100×; scale bar 200 µm (***a***,***B***); magnification 400×; scale bar 100 µm (***c****–**J***)).

**Figure 13:**
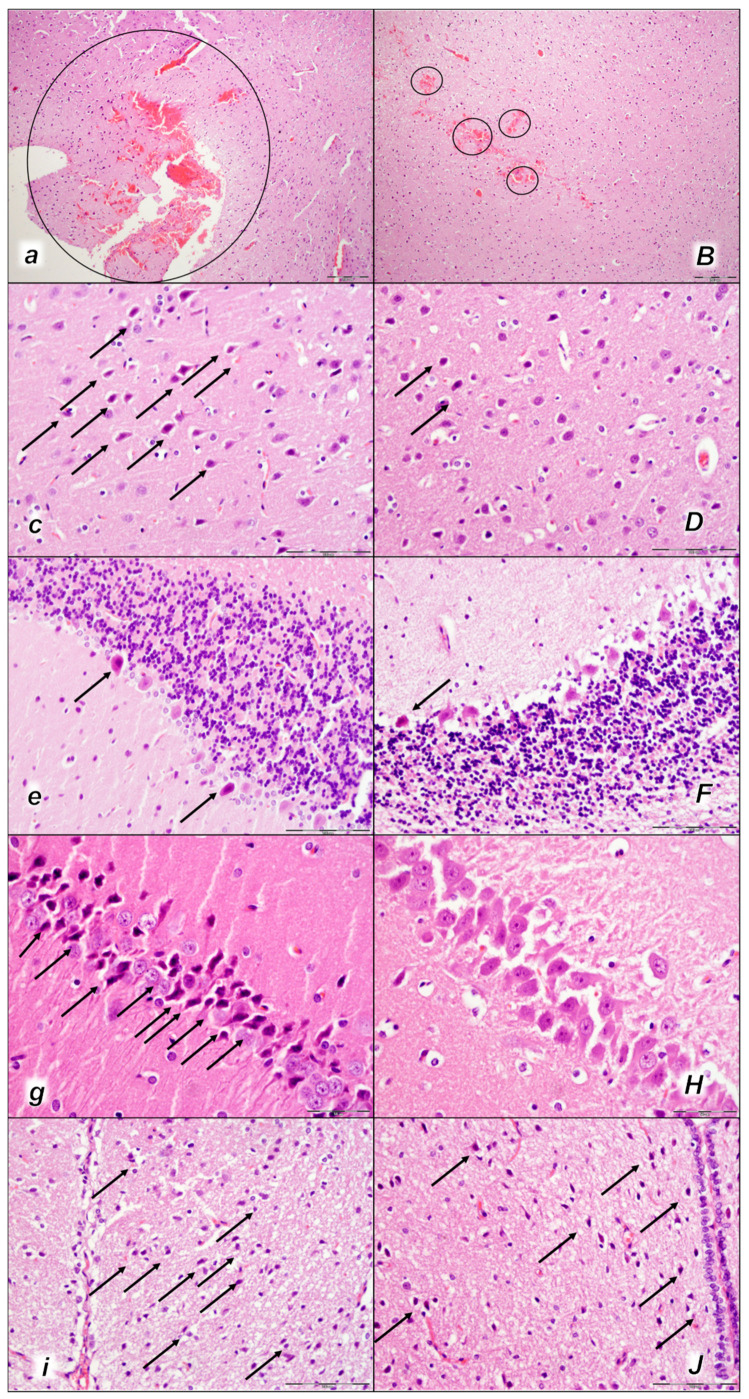
Brain neuropathological changes 60 min following laurate application into the inferior caval vein (***a****–**J***). In the control rats (small italic letters), a pronounced edema and congestion in the brain tissue were observed. Focal, pronounced, and deep intracerebral hemorrhage involving areas of brain tissue was observed, affecting areas of the neocortex, the corpus callosum, the amygdala, and the striatum in the brain tissue (***a***) (marked area). Moderate and severe neurodegenerative changes of the central nervous system, such as rare karyopyknotic cells affecting the cerebral (***c***) and cerebellar (***e***) cortex, a karyopyknosis and degeneration of the Purkinje cells of the cerebellar cortex, and karyopyknosis of cortical neurons, were observed (black arrows), as well as karyopyknosis of cortical neurons, hypothalamic neurons (***g***), and pyramidal cells of the hippocampus (***i***) (black arrows). In the BPC 157-treated rats (capital italic letters), mild edema and congestion in the brain tissue were observed. Intracerebral hemorrhage was visible only within superficial layers of the neocortex (marked area) (***B***). BPC 157-treated rats presented no or only rare karyopyknotic cells in all four regions: the cerebrum (***D***), cerebellum (***F***), hypothalamus/thalamus (***H***), and hippocampus (***J***). ((HE staining; magnification 100×; scale bar 200 µm (***a***,***B***); magnification 400×; scale bar 100 µm (***c****–**J***)).

Indicatively, as a common clue that might be the cause of failure (i.e., intracranial (superior sagittal sinus), portal, caval hypertension, aortal hypotension, progressed thrombosis, peripherally and centrally, failed collateral recruitment, disturbed ECG presentation, peripheral organs lesion) without therapy, all of the laurate-injected rats converged to display similar brain lesions as well. Moreover, this might be a very rapid effect, as seen with the comparative presentation of the brain before laurate application in naive rats, the immediate severe brain swelling upon laurate application, and rapid counteraction immediately upon BPC 157 therapy application (Figure 10). Since the immediate post-application period, there was observable gross brain swelling (Table 2, Figure 10) and increased intracranial (superior sagittal sinus) hypertension (and portal and caval hypertension and aortal hypotension) (Table 1). Microscopically, severe brain edema and congestion as well as large intracerebral hemorrhage in the frontoparietal area consistently occurred (Table 5, Figure 11, Figure 12 and Figure 13). Notably, there is a rapid therapeutic effect observed after BPC 157 application. BPC 157 therapy reduced intracranial (superior sagittal sinus) hypertension and aortal hypotension, and eliminated portal and caval hypertension; additionally, BPC 157 therapy counteracted brain swelling, concurred with only mild brain edema and congestion, lesser brain hemorrhage, and no intraventricular hemorrhage.

#### 2.3.2. Brain Damage

All three assessment time periods (15 min (Table 5, Figure 11), 30 min (Table 5, Figure 12), and 60 min (Table 5, Figure 13) following laurate application showed a pronounced edema and congestion in the brain tissue of the control rats). Pronounced and deep intracerebral hemorrhage involves many brain areas, such as the neocortex, the corpus callosum, the amygdala, and the striatum. Intraventricular hemorrhage was not observed.

In contrast, in the BPC 157-treated rats were less affected, presenting with only mild edema and congestion in the brain tissue and intracerebral hemorrhage only in the superficial layers of the neocortex (15 min (Table 5, Figure 11), 30 min (Table 5, Figure 12), and 60 min (Table 5, Figure 13) following laurate application). No intraventricular hemorrhage was found in treated animals.

After the assessment time period of 15 min, control rats presented mild neurodegenerative changes in the central nervous system; rare karyopyknotic cells affecting the cerebral and cerebellar cortex, a karyopyknosis and degeneration of Purkinje cells of the cerebellar cortex, and karyopyknosis of cortical neurons were observed. In contrast, no neurodegenerative changes of the central nervous system were observed in BPC 157-treated rats (Table 5, Figure 11).

Furthermore, brain lesions rapidly progressed, with control rats severely affected; notably, later than 30 min and 60 min in particular. Moderate and severe neurodegenerative changes were widespread, in the cerebral and cerebellar cortex, hypothalamus/thalamus, and hippocampus, affecting all four regions (Table 5, Figure 12 and Figure 13). There was karyopyknosis and degeneration of the Purkinje cells of the cerebellar cortex, and karyopyknosis of the cortical neurons and pyramidal cells of the hippocampus, as well as hypothalamic neurons. BPC 157-treated rats were much less affected, presenting with no or only rare karyopyknotic cells in all four regions, the cerebral and cerebellar cortex, hypothalamus/thalamus, and hippocampus. Thus, in laurate-administered rats, BPC 157 therapy attenuated/counteracted all inferior caval vein embolization-induced brain lesions.

We suggest the key finding of a particular activated collateral pathway, i.e., the azygos vein, which combined the inferior caval vein and left superior vein to reorganize blood flow. It is likely that direct blood flow delivery via the azygos vein might be responsible for the noted beneficial effects. In summary, BPC 157 therapy attenuated/reversed thrombosis and eliminated pulmonary thromboemboli occluding lung vessels, reversed hemorrhage (brain, lung) possibly along with reversed Virchow triad circumstances, and, as noted before [8,9,10,11,12,13,14,15,16,17,18,19,20,21,22,23,24], counteracted particularly severe occlusion/occlusion-like syndrome. Prime lung lesions and pulmonary thromboemboli were annihilated, venous and arterial thrombosis were markedly attenuated, both peripherally and centrally, combined with markedly reduced/eliminated brain, heart, lung, liver, kidney, and gastrointestinal lesions. After therapy, laurate-administered rats exhibited no portal hypertension and no caval hypertension, and intracranial (superior sagittal sinus) hypertension was markedly attenuated. Aortal hypotension was ameliorated. Bradycardia was attenuated. After BPC 157 therapy, laurate-injected rats exhibited no prolonged PQ interval and QTc interval. In addition, BPC 157 therapy, given in any of the regimens (µg, ng, intraperitoneal, intragastric) supports these effects.

## 3. Discussion

Stable gastric pentadecapeptide BPC 157 therapy, given intraperitoneally or intragastrically in rats after inferior caval vein embolization by sodium laurate application, can provide particular therapy to reverse life-threatening circumstances such as prime lung lesions and thromboemboli occluding lung vessels. Moreover, BPC 157 therapy may reverse rapid post-embolization syndrome otherwise progressing with vascular and multiorgan failure (i.e., brain, heart, lung, liver, kidney, and gastrointestinal lesions) and almost annihilate widespread thrombosis, peripherally and centrally, in arteries and veins. Likewise, it may eliminate/attenuate blood pressure disturbances (intra-cranial (superior sagittal sinus), portal, caval hypertension, and aortal hypotension) as a highlight of completely counteracted otherwise-advanced occlusion/occlusion-like syndrome and Virchow triad circumstances (endothelium lesion, hypercoagulability, stasis). Given the prime lung lesions, luminal occlusion of medium and small lung vessels by thromboemboli, and laurate embolization migration, the counteraction may be more demanding and complex than occlusion/occlusion-like syndromes induced by the occlusion of peripheral [8,9,10,11,12,13,14] and central [15,16] major vessels, and similar noxious procedures [17,18,19,20,21,22,23,24], which were resolved as a whole by BPC 157 therapy [8,9,10,11,12,13,14,15,16,17,18,19,20,21,22,23,24]. Therefore, the advanced effect of the BPC 157 therapy may be due to the complete absence of thromboemboli that otherwise occlude lung vessels, the fully counteracted thickening of the alveolar membranes due to capillary congestion, pulmonary edema, and the dilatation of larger blood vessels, likely also via activated collateral pathways. This may also be via the azygos vein, as direct blood flow delivery [8,9,10,11,12,13,14,15,16,17,18,19,20,21,22,23,24] might counteract all harmful effects that might be produced directly via the intravenous administration of sodium laurate in the inferior caval vein, as a soap embolus, and may also counteract general disturbances (for review, see, i.e., [8,9,10,11,12,13,14,15,16,17,18,19,20,21,22,23,24]). This large counteracting potential, in general, and the activation of the azygos vein rescuing pathway and direct blood flow delivery, in particular, may both be attributed to BPC 157’s special cytoprotective (stomach) background. Conceptually, beneficial pleiotropic therapeutic effects, Robert’s epithelium protection [30], and Szabo’s endothelium protection [31] were further extended to upgraded minor vessels, compensating for failed major vessels, activating collateral pathways and reestablishing reorganized blood flow [6,29]. Likewise, the used protocol, consistently effective as in previous occlusion/occlusion-like syndromes [8,9,10,11,12,13,14,15,16,17,18,19,20,21,22,23,24] (µg- and ng-doses range whether given via intragastric or intraperitoneal application), support its effectiveness and high therapeutic activity in these aggravated conditions as well.

Furthermore, BPC 157 therapy might be a therapy for pulmonary hypertension and right-heart dysfunction in monocrotaline-administered rats, as both a prophylaxis and late therapy [60]; moreover, severely affected lung lesions were resolved in occlusion/occlusion-like syndrome therapy [8,9,10,11,12,13,14,15,16,17,18,19,20,21,22,23,24]. Resolving pulmonary embolism and lung injury as a prime target, and heart failure (i.e., pronounced congestion and dilatation of coronary arteries and their intramyocardial branches up to subendocardial area fully counteracted, progressing extreme bradycardia markedly attenuated), might resolve thereby, multiorgan failure, brain, heart, lung, liver, kidney, and gastrointestinal lesions. Indicatively, there was running competition with the embolization-induced vascular failure, and its particular producing of the most severe threat, which might be an additional particular way to how the variety of the noxious events created the even more severe occlusion/occlusion-like syndrome [8,9,10,11,12,13,14,15,16,17,18,19,20,21,22,23,24]. As such, progressing embolization as the initial cause would additionally specify the already known major causes of occlusion/occlusion-like syndrome [8,9,10,11,12,13,14,15,16,17,18,19,20,21,22,23,24]. There was a variety of the used agents (i.e., alcohol [17], lithium [18], dopamine agonists and antagonists [24], acting peripherally or centrally, beta-agonist [19], beta-antagonist [23], and antiarrhythmic [23]), and a variety of receptors being stimulated or blockaded was consequently involved. Likewise, there was a variety of other procedures, including mechanical compression [21], bile duct occlusion [20], organ perforation [22], and peripheral [8,9,10,11,12,13,14], central [15,16], artery [9,12,13,16], and vein [8,9,10,11,12,14,15] occlusion. Together, progressing embolization acknowledges the evidenced variety of causes [8,9,10,11,12,13,14,15,16,17,18,19,20,21,22,23,24] showing that they may all fairly illustrate the complexity of the advanced occlusion/occlusion-like syndrome and the therapeutic significance of the activated “bypassing key” (azygos way pathway) consistently reported with BPC 157 therapy [8,9,10,11,12,13,14,15,16,17,18,19,20,21,22,23,24].

Thus, it might be the case that with the inferior caval vein embolization also, as a particular rapidly progressing threat, the particular resolving activation of the collateral pathways started immediately upon therapy application. Relayed on the given injury, the azygos vein appears to act as a rapidly upgraded minor vessel and can take over the function of the disabled major vessel, resolving the progressing Virchow triad circumstances in post-embolization syndrome as well by direct blood flow delivery to the superior caval vein, compensating for the vascular failure and reorganizing blood flow [8,9,10,11,12,13,14,15,16,17,18,19,20,21,22,23,24]. Grossly, reporting therapeutic effects, the reversal of a failed collapsed azygos vein into an activated azygos vein and the direct blood flow delivery occurred instantly. Immediate recovery appeared along with activation via the azygos vein and activation of the inferior–superior caval vein-rescuing pathway, and the congested inferior caval vein and superior mesenteric vein were also reversed to their normal vein appearance. Illustratively, as before [8,9,10,11,12,13,14,15,16,17,18,19,20,21,22,23,24], as part of the functioning compensation pathway(s), intracranial (superior sagittal sinus) hypertension was counteracted (i.e., meaning simultaneous reversal of the harmful incapability to drain venous blood adequately for a given cerebral blood inflow without raising venous pressures) and brain swelling grossly rapidly attenuated, evidencing rapid counteraction of such venous and intracranial hypertension. Likewise, acting peripherally and centrally, BPC 157 counteracted portal, caval, and aortal hypotension [8,9,10,11,12,13,14,15,16,17,18,19,20,21,22,23,24]. With the counteracted stasis, the recovery included almost annihilated venous and arterial thrombosis, otherwise progressing peripherally and centrally, and fully counteracted pulmonary thromboemboli. Therefore, BPC 157’s counteracting effects of sodium laurate intravenous administration could make it a common efficacious therapy for resolving vascular injuries in rats [8,9,10,11,12,13,14,15,16,17,18,19,20,21,22,23,24], including those initiated with prime pulmonary lesions.

A common successful result might be the encountered combined evidence. As mentioned, it might be with the BPC 157 therapy the combined chain of the tightly interconnected subsequent events, long ago recognized as an immediate part of the innate activity of the cytoprotective agent [31,32], the maintained cytoprotection endothelium function (for review see, i.e., [1,2,3,4,5,6,25,29]) leading to the BPC 157 activation of the collateral pathways, “bypassing vascular key” [8,9,10,11,12,13,14,15,16,17,18,19,20,21,22,23,24]. This may be the induced NO-release of its own [36,37,38,39] as the interaction with or the modulation of the entire NO-system, as the counteraction of both the NO-synthase blockade (L-NAME-hypertension counteracted) and NOS-substrate over-activity (L-arginine-hypotension counteracted) [38] might be both specifically allocated. In addition, there was the specifically maintained thrombocytes function (i.e., the counteracted L-NAME-pro-thrombotic effect, counteracted L-arginine-anti-thrombotic effect) [32], given that the coagulation pathways were not affected as demonstrated in aggregometry and thromboelastometry studies [32,33,34,35]. Illustratively, BPC 157 given with aspirin, clopidogrel, or cilostazol might specifically maintain the function of thrombocytes activated by arachidonic acid, adenosine diphosphate, collagen, and arachidonic acid/prostaglandin E1 [35]. In addition, providing strong interrelations between the arrhythmias (evidently, this includes also all of the ECG disturbances attenuated [8,9,10,11,12,13,14,15,16,17,18,19,20,21,22,23,24]), heart failure, and thrombosis [8], assuming that the venous and arterial thrombosis are two aspects of the same disease [8], BPC 157’s counteracting effect might be reciprocally related. It may have modulatory effects on the NO-system as a whole (i.e., NO-release, NOS-inhibition, NO-over-stimulation all being affected) (for review, see i.e., [36,37]), and without the need for other known ligands or shear stress, BPC 157 might activate the VEGFR2-Akt-eNOS signaling pathway [40] and maintain the vasomotor tone through the activation of the Src-Caveolin-1-eNOS pathway [41]. Also, these might occur along with modulatory effects on the prostaglandins-system [27,28]. BPC 157 counteracted the toxicity of non-steroidal anti-inflammatory drugs (NSAIDs) (for review, see [61]), counteracted indomethacin-induced leaky gut syndrome (for review, see [4]), and counteracted prolonged bleeding and thrombocytopenia [32,33,34,35] (for review, see also [5,6,8]), in particular. Note that BPC 157 counteracted thrombocyte consumption [8]. Therefore, counteracted thrombosis, prolonged bleeding, and thrombocytopenia in deep vein thrombosis as well [8], besides the reversed Virchow circumstances, might be taken as evidence of BPC 157’s particular wound healing capabilities as being due to cytoprotective agents’ essential effects, realizing the healing process for ruptured blood vessels as whole [2], given the innate distinctive effect on all four major events in clot formation and dissolution. This might be used in distinctive ways depending on the given injury and agent application likewise in the BPC 157-treated rats with vessel occlusion or occlusion-like syndromes [8,9,10,11,12,13,14,15,16,17,18,19,20,21,22,23,24] and, consistently, BPC 157-treated rats with laurate embolization might exhibit almost annihilated thrombosis, peripherally and centrally, and no or markedly attenuated organ hemorrhaging, and, in particular, counteracted brain hemorrhage. This might be common evidence and BPC 157 therapy might provide a dual central/peripheral benefit, since it was effective in either circumstance [25], as specifically shown even in the worst circumstances of intra-abdominal hypertension of grade III and grade IV [21]. With the three body cavities interconnected through the venous system, the disturbances are rapidly transmitted both from the periphery to the centre and from the centre to the periphery, as might be the case with the inferior caval vein embolization. There, while extreme bradycardias would otherwise occur regularly, BPC 157’s therapeutic effect stems from the upgraded venous system (i.e., activated azygos vein) and maintained heart function, and BPC 157-treated rats might smoothly sustain increased intra-abdominal hypertension, of even grade III and grade IV [21], and the inferior caval vein embolization and full reversal of an otherwise downhill course of occlusion/occlusion-like syndrome. Moreover, this may be a fully controlled response associated with its function as a stabilizer of the cellular junction [4], leading to significantly mitigated leaky gut syndrome, via increasing tight junction protein ZO-1 expression and transepithelial resistance [4]. Likewise, there is also the inhibition of the mRNA of inflammatory mediators (iNOS, IL-6, IFN, and TNF-alpha) and the increased expression of HSP 70 and 90 and antioxidant proteins, such as HO-1, NQO-1, glutathione reductase, glutathione peroxidase 2, and GST-pi [4]. Note that BPC 157 is acting also as a free radical scavenger [4,62,63,64,65,66], in vascular failure studies in particular [8,9,10,11,12,13,18,19]). Likewise, a fully controlled response may be associated, for instance, with the counteraction of tumor-induced cachexia and the inhibition of catabolic pathways (IL-6, TNF-alpha) balanced with the stimulation of anabolic pathways (FoxO3a, p-AKT, p-mTOR, and P-GSK-3β) [42].

In the end, these might be taken as an effective upgrade of the cytoprotection maxim endothelium maintenance → epithelium maintenance (for review, see i.e., [6,25,29]) as a powerful cytoprotective agent rapidly acting to recruit collateral pathways.

In conclusion, when using laurate application into the inferior caval vein to cause severe prime lung lesions, thromboemboli occluding lung vessels, severe vascular and multiorgan failure, and particular severe occlusion/occlusion-like-syndrome, BPC 157 therapy provides a clear resolution. The bypassing of the defect as the particular action (i.e., via activation of the azygos vein direct blood flow delivery) organizes blood flow re-establishment and reorganization to compensate for vascular defects and/or reverse induced failure [8,9,10,11,12,13,14,15,16,17,18,19,20,21,22,23,24]. In particular, there was a strong counteraction of the prime lung lesion, thrombosis, and pulmonary thromboemboli. Consistently, it might be rapidly operative in the threatening conditions following embolization, in particular.

## 4. Materials and Methods

### 4.1. Animals

Twelve-week-old male Albino Wistar rats with 200 g body weight, bred in-house at the Animal Pharmacology Facility, School of Medicine, Zagreb, Croatia (registered with the Veterinary Directorate (Reg. No: HR-POK-007)), randomly assigned at six rats/group/interval, were used in all experiments. Rats were acclimated for five days and randomly assigned to their respective treatment groups; housed in polycarbonate (PC) cages (identified with dates, number of study, group, dose, number, and sex of each animal) at 20–24 °C, relative humidity of 40–70%, noise level 60 dB, 12 h of illumination per day (fluorescent lighting), and standard good laboratory practice (GLP) diet and fresh water ad libitum. Procedures were consistent with the standard operating procedures (SOPs) of the Animal Pharmacology Facility and the European Convention for the Protection of Vertebrate Animals used for Experimental and other Scientific Purposes (ETS 123). This study was approved by the local Ethics Committee. Ethical principles of the study complied with the European Directive 010/63/E, the Law on Amendments to the Animal Protection Act (Official Gazette 37/13), the Animal Protection Act (Official Gazette 135/06), the Ordinance on the protection of animals used for scientific purposes (Official Gazette 55/13), the Federation of European Laboratory Animal Science Associations (FELASA) recommendations, and the recommendations of the Ethics Committee of the School of Medicine, University of Zagreb. The experiments were assessed by observers blinded with regards to the treatment.

### 4.2. Drugs

Stable gastric pentadecapeptide BPC 157 (GEPPPGKPADDAGLV, molecular weight 1419; Diagen, Ljubljana, Slovenia), a partial sequence of the human gastric juice protein BPC, which is freely soluble in water at pH 7.0 and in saline, was prepared as a peptide with 99% high-performance liquid chromatography (HPLC) purity, with 1-des-Gly peptide being the main impurity. The BPC 157 dose and application regimens (10 µg or 10 ng/kg given as an intragastric administration or continuously per-orally, in drinking water), were as described previously (i.e., without the use of a carrier or peptidase inhibitor) (for review see, i.e., [8,9,10,11,12,13,14,15,16,17,18,19,20,21,22,23,24]). Sodium laurate was commercially purchased (Sigma, Aldrich, St. Louis, MO, USA) and prepared as described [52,53].

### 4.3. Experimental Protocol

In deeply anesthetized rats (intraperitoneal (ip), 40 mg/kg thiopental (Rotexmedica, Trittau, Germany) and 10 mg/kg diazepam (Apaurin; Krka, Novo Mesto, Slovenia)) were injected, complete calvariectomy was performed, and to induce rapid vascular failure and concomitant general syndrome, we applied 0.1 mL of sodium laurate (10 mg/kg) into the inferior caval vein, with assessment at 15 min, 30 min, and 60 min.

For assessment at 15 min, 30 min, and 60 min, rats received therapy with BPC 157 (10 µg or 10 ng/kg) or saline (5 mL/kg) (controls) as an early intraperitoneal regimen at 5 min upon laurate administration. For assessment at 15 min, rats received BPC 157 or saline (5 mL/kg) as an intragastric administration at 5 min after laurate injection.

After a complete calvariectomy, recordings of brain swelling (before the procedure, after laurate, after therapy application, and before sacrifice) followed the procedure previously used in our vascular studies [8,9,10,11,12,13,14,15,16,17,18,19,20,21,22,23,24]. The calvariectomy procedure included, medially to the superior temporal lines and temporalis muscle attachments, 6 burr holes drilled in three horizontal lines (just basal from the posterior interocular line (two rostral burr holes); just rostral to the lambdoid suture (and transverse sinuses) on both sides (two basal burr holes); in line between the basal and rostral burr holes (two middle burr holes)).

Rats were laparatomized again before sacrifice for the corresponding presentation of the peripheral vessels (azygos vein, superior mesenteric vein, portal vein, inferior caval vein) and corresponding organ lesions (i.e., acute pancreatitis, stomach lesion). The recording was performed with a camera attached to a VMS-004 Discovery Deluxe USB microscope (Veho, USA) at the end of the experiment, and assessed as before [8,9,10,11,12,13,14,15,16,17,18,19,20,21,22,23,24].

### 4.4. Superior Sagittal Sinus, Portal, and Caval Vein, and Abdominal Aorta Pressure Recording

Recordings followed the procedure used and described in detail in our previous vascular studies [8,9,10,11,12,13,14,15,16,17,18,19,20,21,22,23,24]: deeply anesthetized rats, a cannula (BD Neoflon™ Cannula) connected to a pressure transducer (78534C MONITOR/ TERMINAL; Hewlett Packard, Palo Alto, CA, USA), inserted into the portal vein, inferior caval vein, and superior sagittal sinus, as well as the abdominal aorta at the level of the bifurcation at 15 min, 30 min, and 60 min after laurate administration. The superior sagittal sinus’s anterior part was cannulated using a Braun intravenous cannula, then, after laparotomy, a pressure recording in the portal vein, inferior vena cava, and abdominal aorta was performed.

According to our procedure [8,9,10,11,12,13,14,15,16,17,18,19,20,21,22,23,24], a superior sagittal sinus pressure of −24 to −27 mmHg, portal pressure of 3–5 mmHg similar to that of the inferior vena cava (though with values at least 1 mmHg higher in the portal vein), and abdominal aorta blood pressure values of 100–120 mm Hg at the level of the bifurcation were considered as normal in healthy rats.

### 4.5. ECG Recording

ECGs were recorded continuously in deeply anesthetized rats for all three main leads, by positioning stainless steel electrodes on all four limbs using an ECG monitor with a 2090 programmer (Medtronic, Minneapolis, MN, USA) connected to a Waverunner LT342 digital oscilloscope (LeCroy, Chestnut Ridge, NY, USA) (before procedure, at 15 min, 30 min and 60 min after laurate injection before sacrifice). This arrangement enabled precise recordings, measurements, and analysis of ECG parameters [8,9,10,11,12,13,14,15,16,17,18,19,20,21,22,23,24].

### 4.6. Thrombus Assessment

Following sacrifice, the superior sagittal sinus and, peripherally, the portal vein, inferior caval vein, and abdominal aorta were removed from the rats, and the clots were weighed [8,9,10,11,12,13,14,15,16,17,18,19,20,21,22,23,24]. Likewise, to determine the presentation or no presentation of the clots, aorta and inferior caval vein samples were cut serially at a thickness of 5 μm, stained with hematoxylin and eosin, and analyzed in a blinded fashion as described before [33].

### 4.7. Brain, Heart, Vessel, and Volume Presentation

The applied procedure has been used before in our previous vascular studies [8,9,10,11,12,13,14,15,16,17,18,19,20,21,22,23,24]. Brain volume, vessel volume, and heart volume were proportional to the change in the brain, vessel, or heart surface area, respectively. The presentation of the brain and peripheral vessels (superior mesenteric vein, inferior caval vein, azygos vein, and abdominal aorta) was recorded in deeply anesthetized rats, with a camera attached to a VMS-004 Discovery Deluxe USB microscope (Veho, Claymont, DE, USA) [8,9,10,11,12,13,14,15,16,17,18,19,20,21,22,23,24]. The border of the brain (or vessels, or heart) in the image was marked using ImageJ software and then the surface area of the brain (or veins, or heart) was measured. This was carried out with brain (or veins, or heart) images for healthy rats, and then for both the control (saline) group and treated (BPC 157) group of rats at the same intervals after the application and at the time of sacrifice. The arithmetic mean of the surface areas was calculated for both groups. Then, the ratio of these two areas was calculated as ($\frac{A_{con}}{A_{bpc}}$), where Acon is the arithmetic mean brain (or veins, or heart) area of the control group and Abpc is the arithmetic mean brain (or veins, or heart) area of the treated group. Starting from the square–cube law equations *[1]* *[2]*, an equation for the change in brain (or veins, or heart) volume proportional to the change in brain (or veins, or heart) surface area *[6]* was derived. In expressions *[1–5]*, *l* is defined as any arbitrary one-dimensional length of the brain (for example, the rostro-caudal length of the brain), used only for defining the one-dimensional proportion (l2/l1) between two observed brains (or veins, or hearts) and as an inter-factor (and because of that not measured *[6]*) for deriving the final expression *[6]*. The procedure was as follows: $=\times {\left(\frac{l_{2}}{l_{1}}\right)}^{2}$ *[1]* (square-cube law), $=\times {\left(\frac{l_{2}}{l_{1}}\right)}^{3}$ *[2]* (square-cube law), = *[3]* (from *[1]*, after dividing both sides by A1), = *[4]* (from *[3]*, after taking the square root of both sides), = *[5]* (from *[2]*, after dividing both sides by V1), = *[6]* (after incorporating expression *[4]* into equation *[5]*).

### 4.8. Gross Assessment of Gastrointestinal Lesions

For recording, we used a camera attached to a VMS-004 Discovery Deluxe USB microscope (Veho, Claymont, DE, USA)). As described before, gross lesions in the gastrointestinal tract and in the stomach (sum of the longest diameters, mm) were assessed in deeply anesthetized rats, laparatomized before sacrifice [8,9,10,11,12,13,14,15,16,17,18,19,20,21,22,23,24].

### 4.9. Microscopy

As described in our previous studies [8,9,10,11,12,13,14,15,16,17,18,19,20,21,22,23,24], evaluation was carried out by light microscopy using an Olympus 71 digital camera and an Olympus BX51 microscope (OLYMPUS Europa SE&CO.KG). Digital images were saved as uncompressed 24-bit RGB TIFF files using the software program AnalySIS (Olympus Soft Imaging System GmbH, Munster, Germany). Representative tissue specimens (i.e., the brain, liver, kidney, lungs, and heart taken at the end of the experiment, fixed in 10% neutral buffered formalin (pH 7.4) at room temperature for 24 h) were embedded in paraffin, sectioned at 4 μm, stained with hemalaun and eosin (H&E).

#### 4.9.1. Brain Histology

As described in our previous studies [8,9,10,11,12,13,14,15,16,17,18,19,20,21,22,23,24], the brain was dissected according to NTP-7, at Level 3 and 6 with neuroanatomic subsites presented in certain brain sections using coronal sections with three mandatory sections. We used a semiquantitative neuropathological scoring system, and the sum of analyzed affected areas (0–4) (i) and karyopyknotic cells in the brain areas (0–4) (ii) making (i) + (ii) a combined score (0–8), as follows: (i). Specifically affected brain areas (cerebral cortex (NTP-7, Level 3), cerebellar cortex (NTP-7, Level 6), hippocampus, thalamus, and hypothalamus (NTP-7, Level 3)), were scored (0–4), (a score of 0 indicates no histopathologic change), as follows. Small, patchy, complete or incomplete infarcts (≤10% of the area affected) represented a score of 1. Partly confluent or incomplete infarcts (20–30% of the area affected) represented a score of 2. Large confluent complete infarcts (40–60% of the area affected) represented a score of 3. In the cortex, total disintegration of the tissue in the hypothalamus, thalamus, and hippocampus, i.e., large complete infarcts (˃75% of the area affected), represented a score of 4. (ii). Karyopyknotic cells in the affected brain areas were analyzed (0–4) (a score of 0 indicates no change), including the cerebral cortex (NTP-7, Level 3), cerebellar cortex (NTP-7, Level 6), hippocampus, thalamus, and hypothalamus (NTP-7, Level 3), with scoring categorized as follows: a few karyopyknotic neuronal cells (≤20%) (score of 1); patchy areas of karyopyknotic cells (50%) (score of 2); more extensive karyopyknotic areas (75%) (score of 3); complete infarction (100%) (score of 4). Brain tissue hemorrhage was assessed by estimating the percentage of affected areas. Intraventricular hemorrhage was noted as present or absent.

We also assessed the neuronal pathological changes in acquired digital images saved as uncompressed 24-bit RGB TIFF files in the software program AnalySIS (Olympus Soft Imaging System GmbH, Munster, Germany), performing quantitative analysis of neuronal damage in the karyopyknotic areas. The neurons of the cortical cerebrum, cerebellar region, hippocampus, and hypothalamus were counted in 10 different high-powered fields (HPF, 400×) and 3 to 5 serial sections of each sample were used to carry out the count as described in [67]. The field size was 0.24 μm^2^.

We used four criteria for the estimation of the edema: pale myelin, sieve-like appearance of myelinated areas, dilation of perivascular and pericellular spaces, and vacuolar appearance of the neuropil of gray matter. Edema was graded as heavy, moderate, slight, or no edema (score 0–3) [68].

#### 4.9.2. Lung Histology

The same scoring system as in our previous studies [8,9,10,11,12,13,14,15,16,17,18,19,20,21,22,23,24] was used to grade the degree of lung injury in lung tissue analysis. Each of the features (i.e., focal thickening of the alveolar membranes, congestion, pulmonary edema, intra-alveolar hemorrhage, interstitial neutrophil infiltration, and intra-alveolar neutrophil infiltration) was scored (0–3) as absent (0) or present to a mild (1), moderate (2), or severe (3) degree, and a final histology score was determined.

#### 4.9.3. Renal, Liver, and Heart Histology

The same scoring system as in our previous studies [8,9,10,11,12,13,14,15,16,17,18,19,20,21,22,23,24] was used to grade renal (i.e., the degeneration of Bowman’s space and glomeruli, degeneration of the proximal and distal tubules, vascular congestion, and interstitial edema), liver (i.e., vacuolization of hepatocytes and pyknotic hepatocyte nuclei, activation of Kupffer cells, and enlargement of sinusoids), and heart (i.e., dilatation and congestion of blood vessels within the myocardium and coronary arteries) histology. Each specimen was scored using a scale ranging from 0–3 (0—none; 1—mild; 2—moderate; and 3—severe) for each criterion, and a final histology score was determined (0—none; 1—mild; 2—moderate; and 3—severe).

#### 4.9.4. Gastrointestinal Histology

As in our previous studies [8,9,10,11,12,13,14,15,16,17,18,19,20,21,22,23,24], we used a histologic scoring scale adapted from Chui and coworkers [69] for stomach tissue damage, scoring 0–5 (normal to severe) in three categories (mucosal injury, inflammation, hyperemia/hemorrhage) for a total score of 0 to 15, as described by Lane and coworkers [70]. Illustratively, the assessment included morphologic features of mucosal injury (i.e., different grades of epithelial lifting, villi denudation, and necrosis), inflammation (i.e., focal to diffuse according to lamina propria infiltration or subendothelial infiltration), and hyperemia/hemorrhage (i.e., focal to diffuse according to lamina propria or subendothelial localization).

### 4.10. Statistical Analysis

Statistical analysis was performed by parametric one-way analysis of variance (ANOVA), with the Newman–Keuls post-hoc test or the non-parametric Kruskal–Wallis test and subsequently the Mann–Whitney U test used to compare groups. Values are presented as the mean ± standard deviation (SD) and as the minimum/median/maximum. To compare the frequency difference between groups, the chi-squared test or Fischer’s exact test was used. *p* < 0.05 was considered statistically significant.

## 5. Conclusions

Given the resolved progressing embolization and resolved severely impacted lung, thromboemboli otherwise occluding lung vessels, the BPC 157 regimen may be a resolution of the more severe occlusion/occlusion-like syndrome, advanced Virchow triad circumstances in the multiorgan failure peripherally and centrally [8,9,10,11,12,13,14,15,16,17,18,19,20,21,22,23,24]. Practical applicability means that both intraperitoneal and intragastric regimens (and dose range (µg and ng)) are effective and support each other [8,9,10,11,12,13,14,15,16,17,18,19,20,21,22,23,24] and, therefore, possess high therapeutic efficacy. These results might be due to BPC 157’s modulatory effects on several systems (i.e., NO-system [36,37], prostaglandins [62], dopamine [24]), resulting in a particular interaction with several molecular pathways [4,40,41,42,43,44,45,46,47,48,49]. There, BPC 157 therapy counteracted tumor-induced cachexia [42], acting as a stabilizer of cellular junctions that counteracted leaky gut [4], and exerted its beneficial effects by acting as a free radical scavenger [4,62,63,64,65,66], in particular in vascular occlusion studies [8,9,10,11,12,13,18,19], controlling vasomotor tone and the activation of the Src-Caveolin-1-eNOS pathway [40,41]). It consistently increased capability to function even in the worst circumstances, which may be due to a direct effect on blood vessels, the rapid change in the lipid contents, and protein secondary structure conformation produced instantly by BPC 157 therapy [50].

Conceptually, these were attributed to its particular cytoprotective capabilities [6,25,26,27,28,29], and, therefore, the prompt particular activation of the collateral pathways as a resolving key (activation of the azygos vein direct blood flow delivery). This “bypassing key” was promptly activated when confronted with severe vessel and multiorgan failure occlusion/occlusion-like syndrome [6,25,26,27,28,29], occlusion of one or more major vessels [8,9,10,11,12,13,14], widespread vessel injuries by noxious agents, noxious procedure or mechanical compression [17,18,19,20,21,22,23,24], and embolization as the worst scenario used in the present study that may show the full significance of BPC 157 therapy. Finally, the concept’s verification as the resolution of the multiorgan failure of even deadly occlusion/occlusion-like syndromes by BPC 157 therapy [8,9,10,11,12,13,14,15,16,17,18,19,20,21,22,23,24] goes with the evidence of the in situ hybridization and immunostaining of BPC 157 found in many tissues in both the human fetus and in adults [2,71]. Possibly, BPC 157 may have a regulatory physiologic role in bodily functions, given its similar beneficial effects in other species as well (i.e., birds [72] and insects [73,74]). The final advantage of BPC 157 therapy is also its remarkable safety (i.e., no adverse effects in clinical trials (ulcerative colitis, phase II), and LD1 could be not achieved in toxicological studies) (for review see, i.e., [1,2,3,4,5,6,25,26,27,28,29]), a point recently confirmed in a large study conducted by Xu and collaborators [75]. There is also its easy application (being stable and native in human gastric juice for more than 24 h), and large effectiveness range (10 µg/kg, 10 ng/kg intraperitoneally or intragastrically), both of which supports the effect of the other. Together, after laurate-caused inferior caval vein embolization, resolving prime lung lesions and thromboemboli occluding lung vessels as well as counteracting post-embolization syndrome as a result of particularly severe occlusion/occlusion-like syndrome by BPC 157 therapy may be indicative findings (for review see, i.e., [1,2,3,4,5,6,7,25,26,27,28,29,36,37,61,71]). This may be suggestive of the implementation of the cytoprotection concept and theory [30,31] by BPC 157 therapy’s application to resolve further vascular and embolization injuries.

## Figures and Tables

**Table 1:** Blood pressures and thrombosis in rats at 15 min, 30 min, and 60 min following application of 0.1 mL/rat of sodium laurate (10 mg/kg) into inferior caval vein. Means ± SD, * *p* < 0.05, at least vs. control. * p ˂ 0.05, at least vs. *control*.

	Blood Pressures and Thrombosis in Rats at 15 min, 30 min, and 60 min Following Application of 0.1 mL/rat of Sodium Laurate (10 mg/kg) into Inferior Caval Vein					
Assessment	15 min		30 min		60 min	
Medication Application	Intragastric	Intraperitoneal	Intragastric	Intraperitoneal	Intragastric	Intraperitoneal
	**Superior Sagittal Sinus Pressure, mm Hg, Means ± SD**					
Control	7 ± 1	8 ± 1	8 ± 1	9 ± 1	8 ± 1	7 ± 1
BPC 157 10 μg/kg	−1 ± 1 *	−1 ± 1 *	−1 ± 1 *	−1 ± 1 *	−1 ± 1 *	−1 ± 1 *
BPC 157 10 ng/kg	−1 ± 1 *	−1 ± 1 *	−1 ± 1 *	−1 ± 1 *	−1 ± 1 *	−1 ± 1 *
	**Portal pressure, mm Hg, Means ± SD**					
Control	17 ± 1	19 ± 3	17 ± 3	18 ± 2	18 ± 3	17 ± 2
BPC 157 10 μg/kg	6 ± 2 *	5 ± 2 *	5 ± 1 *	6 ± 1 *	4 ± 1 *	5 ± 1 *
BPC 157 10 ng/kg	6 ± 1 *	5 ± 1 *	4 ± 1 *	5 ± 1 *	5 ± 1 *	4 ± 1 *
	**Caval pressure, mm Hg, Means ± SD**					
Control	10 ± 1	11 ± 1	12 ± 1	13 ± 1	12 ± 2	11 ± 1
BPC 157 10 μg/kg	4 ± 1 *	4 ± 1 *	4 ± 1 *	3 ± 1 *	4 ± 1 *	4 ± 1 *
BPC 157 10 ng/kg	4 ± 1 *	4 ± 1 *	3 ± 1 *	4 ± 1 *	5 ± 1 *	3 ± 1 *
	**Abdominal aorta pressure, mm Hg, Means ± SD**					
Control	70 ± 10	73 ± 12	65 ± 12	60 ± 9	65 ± 11	70 ± 9
BPC 157 10 μg/kg	100 ± 8 *	105 ± 8 *	105 ± 10 *	100 ± 7 *	105 ± 11 *	98 ± 9 *
BPC 157 10 ng/kg	102 ± 9 *	100 ± 10 *	98 ± 11 *	108 ± 11 *	98 ± 8 *	100 ± 11 *
	**Superior sagittal sinus, thrombus mass, g, Means ± SD**					
Control	0.0009 ± 0.0002	0.0013 ± 0.0003	0.0029 ± 0.0009	0.0021 ± 0.0006	0.0041 ± 0.001	0.0049 ± 0.0012
BPC 157 10 μg/kg	0.0002 ± 0.0001 *	0.0001 ± 0.00008 *	0.0006 ± 0.0003 *	0.0005 ± 0.0002 *	0.0003 ± 0.0001 *	0.0006 ± 0.0002 *
BPC 157 10 ng/kg	0.0001 ± 0.00007 *	0.0003 ± 0.00009 *	0.0008 ± 0.0002 *	0.0007 ± 0.0002 *	0.0006 ± 0.0001 *	0.0008 ± 0.0003 *
	**Portal vein, thrombus mass, g, Means ± SD**					
Control	0.0012 ± 0.0002	0.0014 ± 0.0003	0.0035 ± 0.0009	0.0045 ± 0.0010	0.0065 ± 0.0015	0.0058 ± 0.0012
BPC 157 10 μg/kg	0.0005 ± 0.0002 *	0.0006 ± 0.0002 *	0.0013 ± 0.0007 *	0.0015 ± 0.0007 *	0.0005 ± 0.0002 *	0.0002 ± 0.0001 *
BPC 157 10 ng/kg	0.0004 ± 0.0001 *	0.0004 ± 0.0002 *	0.0010 ± 0.0005 *	0.0012 ± 0.0005 *	0.007 ± 0.0003 *	0.0004 ± 0.0002 *
	**Inferior caval vein, thrombus mass, g, Means ± SD**					
Control	0.0021 ± 0.0005	0.0029 ± 0.0007	0.0089 ± 0.001	0.0082 ± 0.0012	0.0282 ± 0.009	0.032 ± 0.008
BPC 157 10 μg/kg	0.0008 ± 0.0002 *	0.0005 ± 0.0002 *	0.0015 ± 0.0009 *	0.0010 ± 0.0005 *	0.0030 ± 0.0008 *	0.0020 ± 0.0009 *
BPC 157 10 ng/kg	0.0006 ± 0.0002 *	0.0007 ± 0.0002 *	0.0018 ± 0.0007 *	0.0015 ± 0.0006 *	0.0028 ± 0.0005 *	0.0025 ± 0.0008 *
	**Abdominal aorta, thrombus mass, g, Means ± SD**					
Control	0.0012 ± 0.0004	0.0015 ± 0.0006	0.0025 ± 0.0007	0.0022 ± 0.0008	0.0066 ± 0.0012	0.0061 ± 0.0014
BPC 157 10 μg/kg	0.0005 ± 0.0002 *	0.0003 ± 0.0001 *	0.0013 ± 0.0006 *	0.0011 ± 0.0005 *	0.0014 ± 0.0006 *	0.0011 ± 0.0004 *
BPC 157 10 ng/kg	0.0004 ± 0.0001 *	0.0005 ± 0.0001 *	0.0011 ± 0.0005 *	0.0013 ± 0.0004 *	0.0010 ± 0.0004 *	0.0015 ± 0.0007 *
	**Superior mesenteric artery, thrombus mass, g, Means ± SD**					
Control	0.0010 ± 0.0004	0.0013 ± 0.0005	0.0033 ± 0.0005	0.0043 ± 0.0009	0.0073 ± 0.0008	0.0083 ± 0.0012
BPC 157 10 μg/kg	0.0004 ± 0.0002 *	0.0003 ± 0.0001 *	0.0013 ± 0.0006 *	0.0011 ± 0.0005 *	0.0016 ± 0.0007 *	0.0010 ± 0.0005 *
BPC 157 10 ng/kg	0.0005 ± 0.0001 *	0.0004 ± 0.0002 *	0.0010 ± 0.0004 *	0.0014 ± 0.0004 *	0.0012 ± 0.0005 *	0.0015 ± 0.0007 *

**Table 2:** Relative volume (control/treated) (%) of the brain, heart, azygos vein, inferior caval vein, superior mesenteric vein, and abdominal aorta in rats at 15 min, 30 min, and 60 min following application of 0.1 mL of sodium laurate (10 mg/kg) into inferior caval vein. * p ˂ 0.05, at least vs. *control*.

	Relative Volume (Control/Treated) (%) of the Brain, Heart, Azygos Vein, Inferior Caval Vein, Superior Mesenteric Vein, and Abdominal Aorta in Rats at 15 min, 30 min, and 60 min Following Application of 0.1 mL of Sodium Laurate (10 mg/kg) into Inferior Caval Vein					
Assessment	15 min		30 min		60 min	
Medication Application	Intragastric	Intraperitoneal	Intragastric	Intraperitoneal	Intragastric	Intraperitoneal
	**Relative Volume (Control/Treated) (%) of the Brain, Means ± SD**					
BPC 157 10 μg/kg	123 ± 7 *	122 ± 7 *	124 ± 7 *	122 ± 6 *	126 ± 7 *	126 ± 5 *
BPC 157 10 ng/kg	120 ± 8 *	124 ± 9 *	123 ± 5 *	124 ± 5 *	123 ± 8 *	124 ± 7 *
	**Relative volume (control/treated) (%) of the heart, Means ± SD**					
BPC 157 10 μg/kg	130 ± 7 *	135 ± 87 *	124 ± 7 *	128 ± 7 *	140 ± 6 *	135 ± 8 *
BPC 157 10 ng/kg	126 ± 87 *	133 ± 7 *	129 ± 8 *	129 ± 9 *	135 ± 5 *	141 ± 7 *
	**Relative volume (control/treated) (%) of the azygos vein, Means ± SD**					
BPC 157 10 μg/kg	25 ± 1 *	11 ± 1 *	33 ± 1 *	13 ± 1 *	47 ± 2 *	11 ± 1 *
BPC 157 10 ng/kg	4 ± 1 *	4 ± 1 *	3 ± 1 *	4 ± 1 *	5 ± 1 *	3 ± 1 *
	**Relative volume (control/treated) (%) of the inferior caval vein, Means ± SD**					
Control	181 ± 10 *	73 ± 12 *	182 ± 12 *	60 ± 9 *	197 ± 11 *	70 ± 9 *
BPC 157 10 μg/kg	*100 ± 8*	*105 ± 8*	105 ± 10 *	100 ± 7 *	105 ± 11 *	98 ± 9 *
BPC 157 10 ng/kg	102 ± 9 *	100 ± 10 *	98 ± 11 *	108 ± 11 *	98 ± 8 *	100 ± 11 *
	**Relative volume (control/treated) (%) of the superior mesenteric vein, Means ± SD**					
BPC 157 10 μg/kg	150 ± 10 *	155 ± 12 *	178 ± 12 *	170 ± 9 *	132 ± 8 *	145 ± 10 *
BPC 157 10 ng/kg	145 ± 12 *	152 ± 9 *	182 ± 14 *	175 ± 11 *	142 ± 9 *	140 ± 8 *
	**Relative volume (control/treated) (%) of the abdominal aorta, Means ± SD**					
BPC 157 10 μg/kg	49 ± 7 *	52 ± 7 *	56 ± 8 *	58 ± 6 *	58 ± 8 *	55 ± 5 *
BPC 157 10 ng/kg	45 ± 5 *	55 ± 5 *	46 ± 5 *	48 ± 5 *	57 ± 6 *	57 ± 7 *

**Table 3:** ECG disturbances in rats at 15 min, 30 min, and 60 min following application of 0.1 mL of sodium laurate (10 mg/kg) into inferior caval vein * p ˂ 0.05, at least vs. *control*.

	ECG Changes in Rats at 15 min, 30 min, and 60 min Following Application of 0.1 mL of Sodium Laurate (10 mg/kg) into the Inferior Caval Vein					
Assessment	15 min		30 min		60 min	
Medication Application	Intragastric	Intraperitoneal	Intragastric	Intraperitoneal	Intragastric	Intraperitoneal
	**PQ Interval, msec Means ± SD**					
Control	60 ± 5	62 ± 5	70 ± 5	73 ± 5	80 ± 5	84 ± 5
BPC 157 10 μg/kg	50 ± 5 *	50 ± 5 *	60 ± 5 *	60 ± 5 *	60 ± 5 *	60 ± 5 *
BPC 157 10 ng/kg	50 ± 5 *	50 ± 5 *	60 ± 5 *	60 ± 5 *	60 ± 5 *	60 ± 5 *
	**QTc interval, msec, Means ± SD**					
Control	350 ± 10	352 ± 12	360 ± 10	363 ± 10	380 ± 10	383 ± 10
BPC 157 10 μg/kg	235 ± 10 *	225 ± 10 *	250 ± 10 *	254 ± 10 *	274 ± 10 *	272 ± 10 *
BPC 157 10 ng/kg	230 ± 10 *	227 ± 10 *	252 ± 10 *	250 ± 10 *	270 ± 10 *	276 ± 10 *
	**Heart frequency, beats/min, Means ± SD**					
Control	100 ± 10	110 ± 10	75 ± 9	70 ± 9	55 ± 5	50 ± 5
BPC 157 10 μg/kg	280 ± 8 *	290 ± 10 *	260 ± 10 *	265 ± 8 *	235 ± 10 *	237 ± 8 *
BPC 157 10 ng/kg	282 ± 9 *	285 ± 10 *	268 ± 11 *	270 ± 9 *	230 ± 10 *	232 ± 11 *

**Table 4:** Lesions were scored microscopically (heart, lung, liver, kidney, gastrointestinal tract) or macroscopically (stomach) in rats at 15 min, 30 min, and 60 min following application of 0.1 mL of sodium laurate (10 mg/kg) into inferior caval vein. * p ˂ 0.05, at least vs. *control*.

	Lesions Scored Microscopically (Heart, Lung, Liver, Kidney) or Macroscopically (Stomach) in Rats at 15 min, 30 min, and 60 min Following Application of 0.1 mL of Sodium Laurate (10 mg/kg) into Inferior Caval Vein					
Assessment	15 min		30 min		60 min	
Medication Application	Intragastrical	Intraperitoneal	Intragastrical	Intraperitoneal	Intragastrical	Intraperitoneal
	**Heart (Scored 0–3, Min/Med/Max)**					
Control	2/3/3	2/3/3	2/3/3	2/3/3	3/3/3	3/3/3
BPC 157 10 μg/kg	0/1/1 *	0/1/1 *	0/1/1 *	0/1/1 *	0/1/1 *	0/1/1 *
BPC 157 10 ng/kg	0/1/1 *	0/1/1 *	0/1/1 *	0/1/1 *	0/1/1 *	0/1/1 *
	**Lung (scored 0–3, Min/Med/Max)**					
Control	2/3/3	2/3/3	3/3/3	3/3/3	3/3/3	3/3/3
BPC 157 10 μg/kg	0/0/0 *	0/0/0 *	0/0/0 *	0/0/0 *	0/0/0 *	0/0/0 *
BPC 157 10 ng/kg	0/0/0 *	0/0/0 *	0/0/0 *	0/0/0 *	0/0/0 *	0/0/0 *
	**Liver (scored 0–3, Min/Med/Max)**					
Control	3/3/3	3/3/3	3/3/3	3/3/3	3/3/3	3/3/3
BPC 157 10 μg/kg	0/0/0 *	0/0/0 *	0/0/0 *	0/0/0 *	0/1/1 *	0/1/1 *
BPC 157 10 ng/kg	0/0/0 *	0/0/0 *	0/0/0 *	0/0/0 *	0/1/1 *	0/1/1 *
	**Kidney (scored 0–3, Min/Med/Max)**					
Control	2/2/2	2/2/2	2/2/2	2/2/2	2/3/3	2/3/3
BPC 157 10 μg/kg	0/0/0 *	0/0/0 *	0/0/0 *	0/0/0 *	0/0/0 *	0/0/0 *
BPC 157 10 ng/kg	0/0/0 *	0/0/0 *	0/0/0 *	0/0/0 *	0/0/0 *	0/0/0 *
	**Stomach (sum of longest diameters, mm, Means ± SD)**					
Control	4 ± 1	4 ± 1	5 ± 1	5 ± 1	6 ± 1	6 ± 1
BPC 157 10 μg/kg	0 ± 0 *	0 ± 0 *	0 ± 0 *	0 ± 0 *	0 ± 0 *	0 ± 0 *
BPC 157 10 ng/kg	0 ± 0 *	0 ± 0 *	0 ± 0 *	0 ± 0 *	0 ± 0 *	0 ± 0 *
	**Stomach (scored 0–15, Min/Med/Max)**					
Control	5/5/5	5/5/5	5/5/5	5/5/5	5/5/5	5/5/5
BPC 157 10 μg/kg	0/0/0 *	0/0/0 *	0/0/0 *	0/0/0 *	0/0/0 *	0/0/0 *
BPC 157 10 ng/kg	0/0/0 *	0/0/0 *	0/0/0 *	0/0/0 *	0/0/0 *	0/0/0 *
	**Small intestine (scored 0–15, Min/Med/Max)**					
Control	5/5/5	5/5/5	5/5/5	5/5/5	5/5/5	5/5/5
BPC 157 10 μg/kg	0/0/0 *	0/0/0 *	0/0/0 *	0/0/0 *	0/0/0 *	0/0/0 *
BPC 157 10 ng/kg	0/0/0 *	0/0/0 *	0/0/0 *	0/0/0 *	0/0/0 *	0/0/0 *
	**Large intestine (scored 0–15, Min/Med/Max)**					
Control	5/5/5	5/5/5	5/5/5	5/5/5	5/5/5	5/5/5
BPC 157 10 μg/kg	0/0/0 *	0/0/0 *	0/0/0 *	0/0/0 *	0/0/0 *	0/0/0 *
BPC 157 10 ng/kg	0/0/0 *	0/0/0 *	0/0/0 *	0/0/0 *	0/0/0 *	0/0/0 *

**Table 5:** Lesions were scored microscopically in the cerebrum, cerebellum, hypothalamus, and hippocampus in rats at 15 min, 30 min, and 60 min following the application of 0.1 mL of sodium laurate (10 mg/kg) into the inferior caval vein. * p ˂ 0.05, at least vs. *control*.

	Lesions Scored Microscopically in the Cerebrum, Cerebellum, Hypothalamus, and Hippocampus in Rats at 15 min, 30 min, and 60 min Following Application of 0.1 mL of Sodium Laurate (10 mg/kg) into the Inferior Caval Vein					
Assessment	15 min		30 min		60 min	
Medication Application	Intragastric	Intraperitoneal	Intragastric	Intraperitoneal	Intragastric	Intraperitoneal
	**Cerebrum (Scored 0–8, Min/Med/Ma) #**					
Control	1/1/1	1/1/1	2/2/2	2/2/2	2/3/3	2/3/3
BPC 157 10 μg/kg	0/0/0 *	0/0/0 *	0/1/1 *	0/1/1 *	0/1/1 *	0/1/1 *
BPC 157 10 ng/kg	0/0/0 *	0/0/0 *	0/1/1 *	0/1/1 *	0/1/1 *	0/1/1 *
	Neuronal damage in the karyopyknotic areas, %, Means ± SD (10 HPF, 400×)					
Control	11 ± 3	13 ± 3	25 ± 3	23 ± 3	26 ± 2	25 ± 2
BPC 157 10 μg/kg	0 ± 0 *	0 ± 0 *	2 ± 1 *	2 ± 1 *	2 ± 1 *	2 ± 1 *
BPC 157 10 ng/kg	0 ± 0 *	0 ± 0 *	2 ± 1 *	2 ± 1 *	2 ± 1 *	2 ± 1 *
	Hemorrhage (% of total area), Means ± SD					
Control	30 ± 3	35 ± 3	32 ± 3	35 ± 3	36 ± 3	35 ± 4
BPC 157 10 μg/kg	3 ± 1 *	3 ± 1 *	3 ± 1 *	3 ± 1 *	3 ± 1 *	3 ± 1 *
BPC 157 10 ng/kg	3 ± 1 *	3 ± 1 *	3 ± 1 *	3 ± 1 *	3 ± 1 *	3 ± 1 *
	Edema (scored 0–3, Min/Med/Max)					
Control	2/3/3	2/3/3	2/3/3	2/3/3	2/3/3	2/3/3
BPC 157 10 μg/kg	0/1/1 *	0/1/1 *	0/1/1 *	0/1/1 *	0/1/1 *	0/1/1 *
BPC 157 10 ng/kg	0/1/1 *	0/1/1 *	0/1/1 *	0/1/1 *	0/1/1 *	0/1/1 *
	**Cerebellum (scored 0–8, Min/Med/Ma)**					
Control	1/1/1	1/1/1	1/1/1	1/1/1	1/1/1	1/1/1
BPC 157 10 μg/kg	0/0/0 *	0/0/0 *	0/0/0 *	0/0/0 *	0/0/0 *	0/0/0 *
BPC 157 10 ng/kg	0/0/0 *	0/0/0 *	0/0/0 *	0/0/0 *	0/0/0 *	0/0/0 *
	Neuronal damage in the karyopyknotic areas, %, Means ± SD (10 HPF, 400×)					
Control	10 ± 2	10 ± 2	10 ± 5	10 ± 2	10 ± 5	10 ± 5
BPC 157 10 μg/kg	0 ± 0 *	0 ± 0 *	2 ± 1 *	2 ± 1 *	2 ± 1 *	2 ± 1 *
BPC 157 10 ng/kg	0 ± 0 *	0 ± 0 *	2 ± 1 *	2 ± 1 *	2 ± 1 *	2 ± 1 *
	Hemorrhage (% of total area)					
Control	0	0	0	0	0	0
BPC 157 10 μg/kg	0	0	0	0	0	0
BPC 157 10 ng/kg	0	0	0	0	0	0
	Edema (scored 0–3, Min/Med/Max)					
Control	2/2/2	2/2/2	2/2/2	2/2/2	2/2/2	2/2/2
BPC 157 10 μg/kg	0/1/1 *	0/1/1 *	0/1/1 *	0/1/1 *	0/1/1 *	0/1/1 *
BPC 157 10 ng/kg	0/1/1 *	0/1/1 *	0/1/1 *	0/1/1 *	0/1/1 *	0/1/1 *
	**Hippocampus (scored 0–8, Min/Med/Max)**					
Control	0/1/1 *	0/1/1 *	3/3/3	3/3/3	3/3/3	3/3/3
BPC 157 10 μg/kg	0/0/0 *	0/0/0 *	0/1/1 *	0/1/1 *	0/1/1 *	0/1/1 *
BPC 157 10 ng/kg	0/0/0 *	0/0/0 *	0/1/1 *	0/1/1 *	0/1/1 *	0/1/1 *
	Neuronal damage in the karyopyknotic areas, %, Means ± SD (10 HPF, 400×)					
Control	11 ± 2	13 ± 2	48 ± 2	50 ± 2	51 ± 3	53 ± 3
BPC 157 10 μg/kg	0 ± 0 *	0 ± 0 *	2 ± 1 *	2 ± 1 *	2 ± 1 *	2 ± 1 *
BPC 157 10 ng/kg	0 ± 0 *	0 ± 0 *	2 ± 1 *	2 ± 1 *	2 ± 1 *	2 ± 1 *
	Hemorrhage (% of total area), Means ± SD					
Control	0	0	0	0	0	0
BPC 157 10 μg/kg	0	0	0	0	0	0
BPC 157 10 ng/kg	0	0	0	0	0	0
	Edema (scored 0–3, Min/Med/Max)					
Control	2/2/2	2/2/2	2/2/2	2/2/2	2/2/2	2/2/2
BPC 157 10 μg/kg	0/1/1 *	0/1/1 *	0/1/1 *	0/1/1 *	0/1/1 *	0/1/1 *
BPC 157 10 ng/kg	0/1/1 *	0/1/1 *	0/1/1 *	0/1/1 *	0/1/1 *	0/1/1 *
	**Hypothalamus (scored 0–8, Min/Med/Max)**					
Control	0/0/0 *	0/0/0 *	1/1/1	1/1/1	1/2/2	1/2/2
BPC 157 10 μg/kg	0/0/0 *	0/0/0 *	0/1/1 *	0/1/1 *	0/1/1 *	0/1/1 *
BPC 157 10 ng/kg	0/0/0 *	0/0/0 *	0/1/1 *	0/1/1 *	0/1/1 *	0/1/1 *
	Neuronal damage in the karyopyknotic areas, %, Means ± SD (10 HPF, 400×)					
Control	0 ± 0	0 ± 0	10 ± 2	10 ± 2	30 ± 3	33 ± 3
BPC 157 10 μg/kg	0 ± 0	0 ± 0	2 ± 1 *	2 ± 1 *	10 ± 2 *	12 ± 2 *
BPC 157 10 ng/kg	0 ± 0	0 ± 0	2 ± 1 *	2 ± 1 *	11 ± 2 *	10 ± 3 *
	Hemorrhage (% of total area), Means ± SD					
Control	0	0	0	0	0	0
BPC 157 10 μg/kg	0	0	0	0	0	0
BPC 157 10 ng/kg	0	0	0	0	0	0
	Edema (scored 0–3, Min/Med/Max)					
Control	2/2/2	2/2/2	2/2/2	2/2/2	2/2/2	2/2/2
BPC 157 10 μg/kg	0/1/1 *	0/1/1 *	0/1/1 *	0/1/1 *	0/1/1 *	0/1/1 *
BPC 157 10 ng/kg	0/1/1 *	0/1/1 *	0/1/1 *	0/1/1 *	0/1/1 *	0/1/1 *

## Data Availability

The data presented in this study are available on request from the corresponding author.
